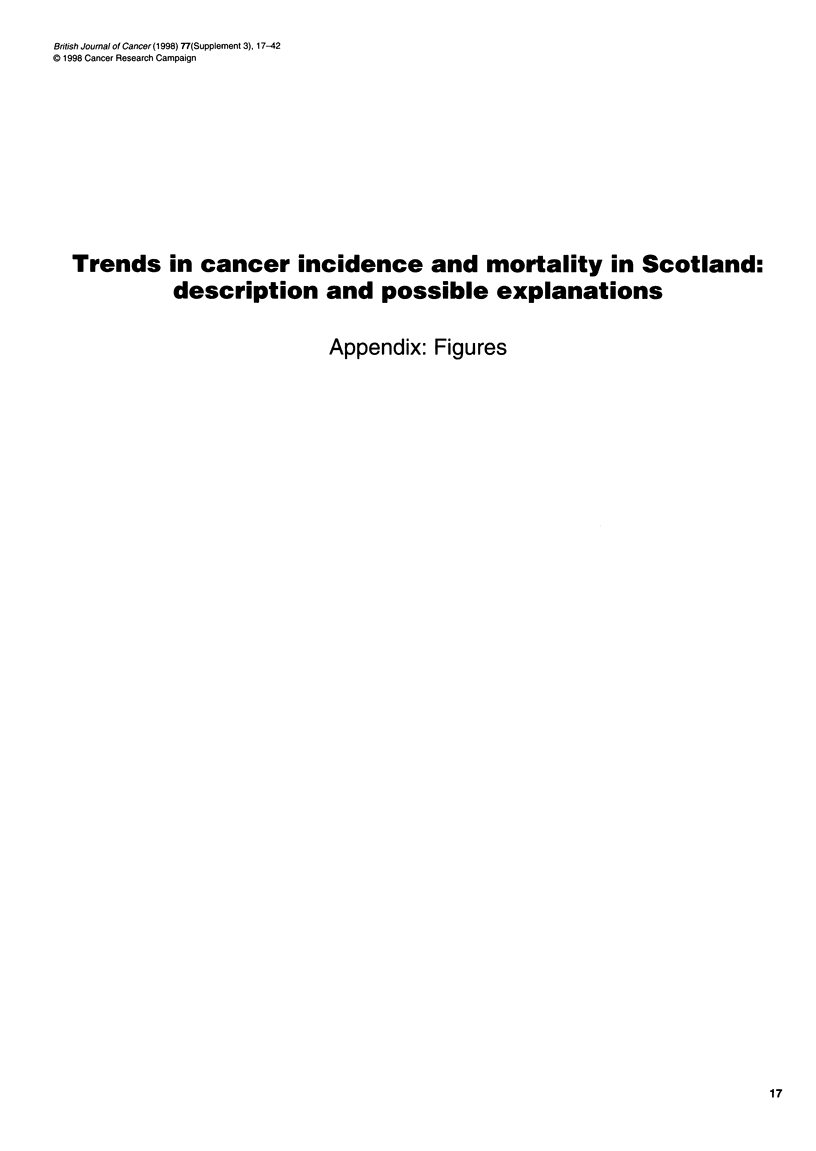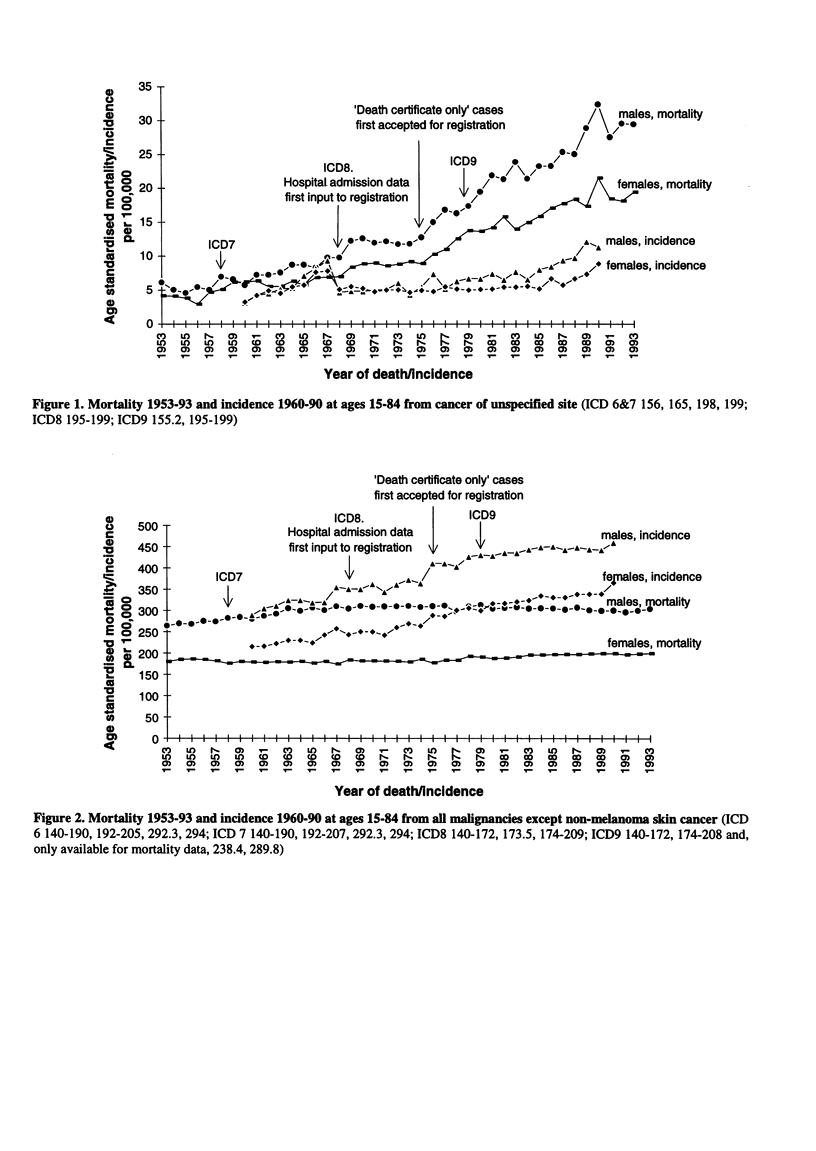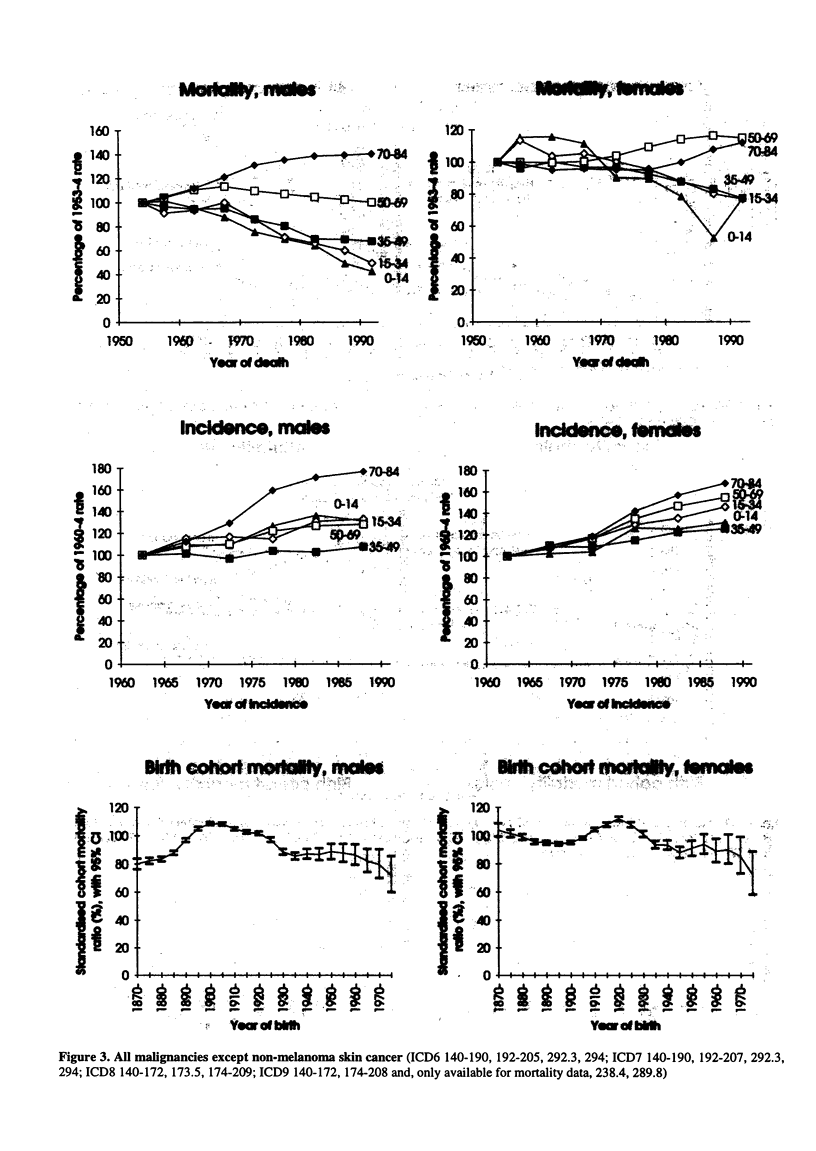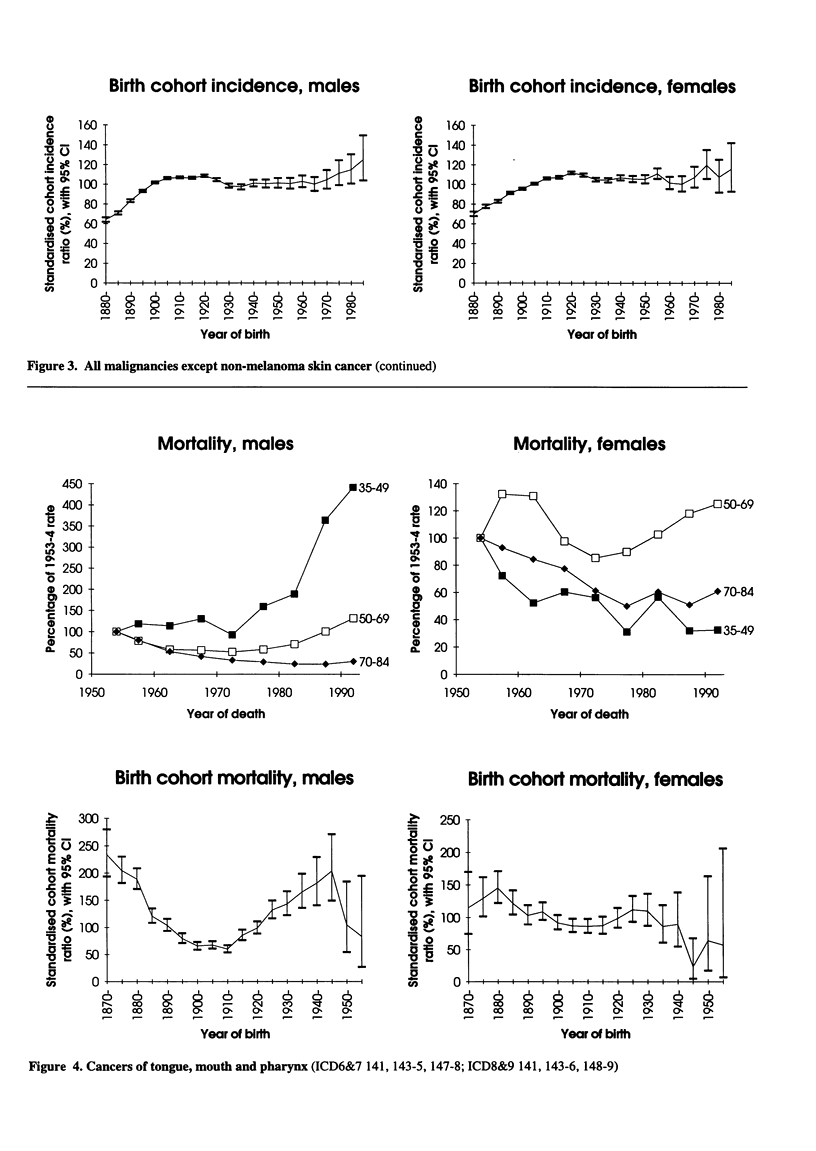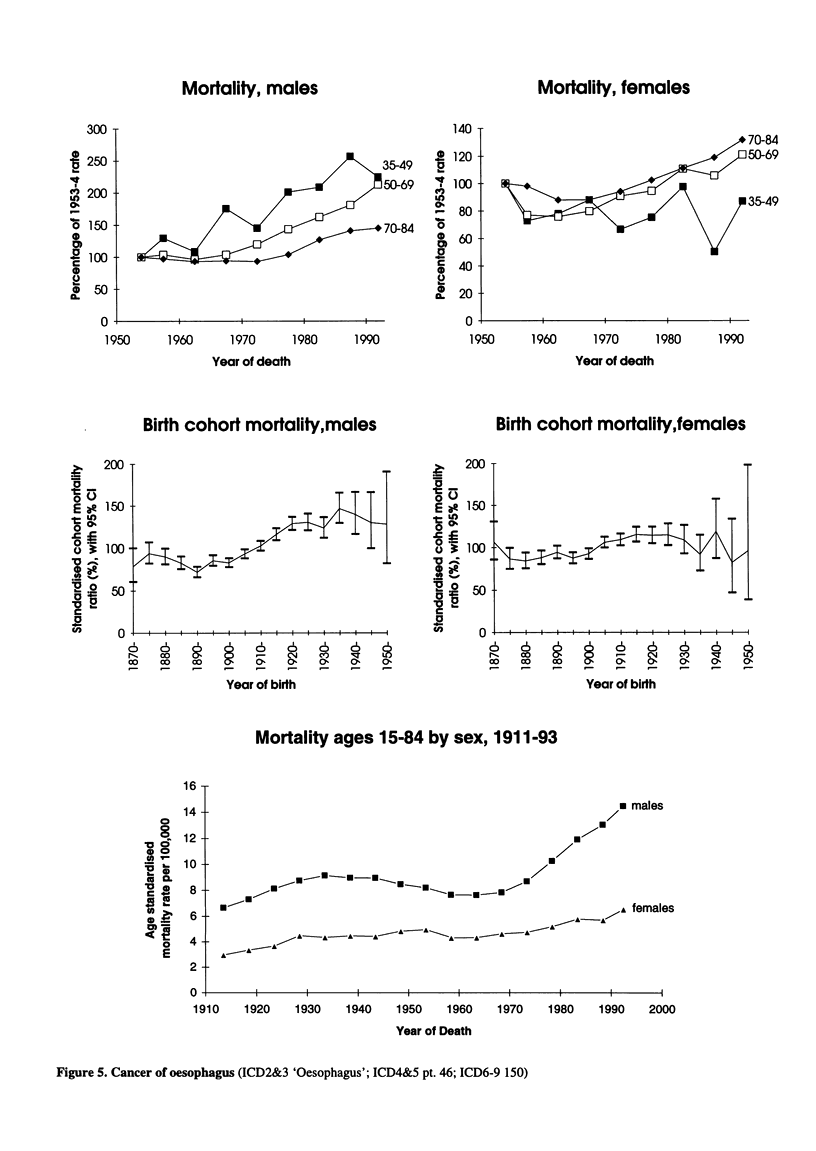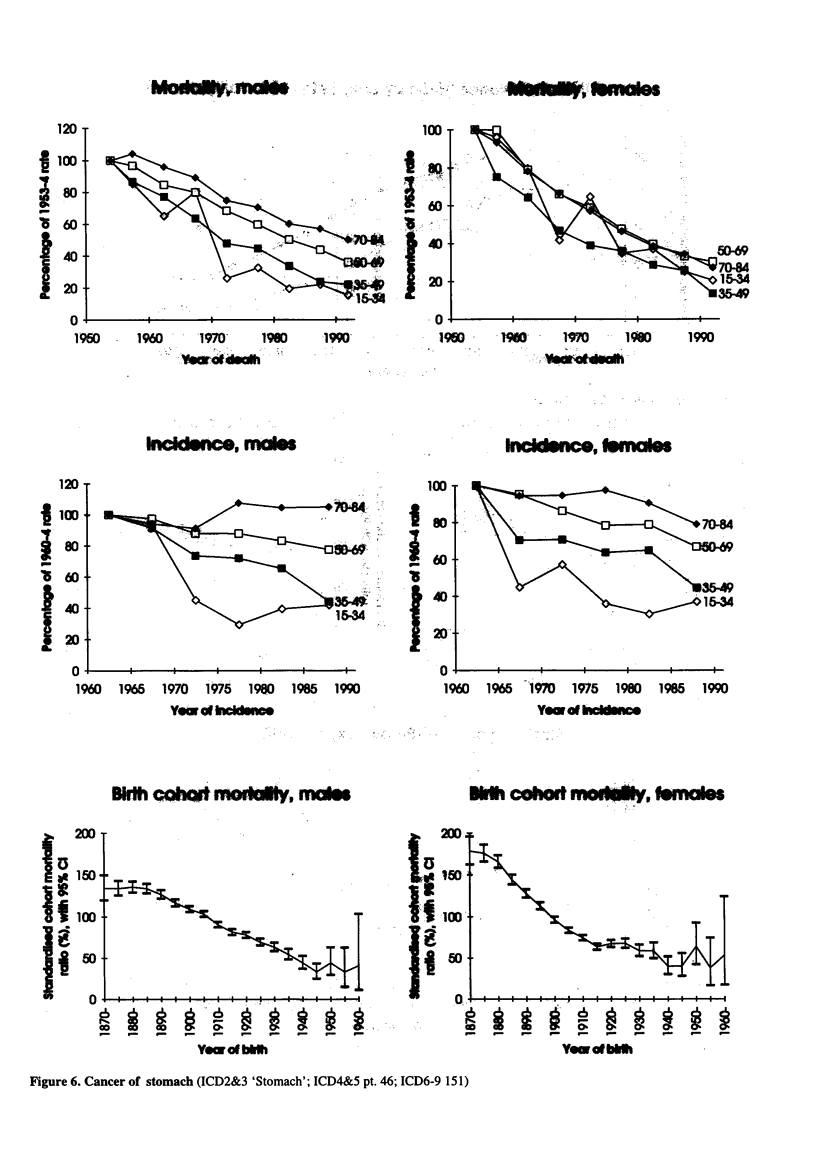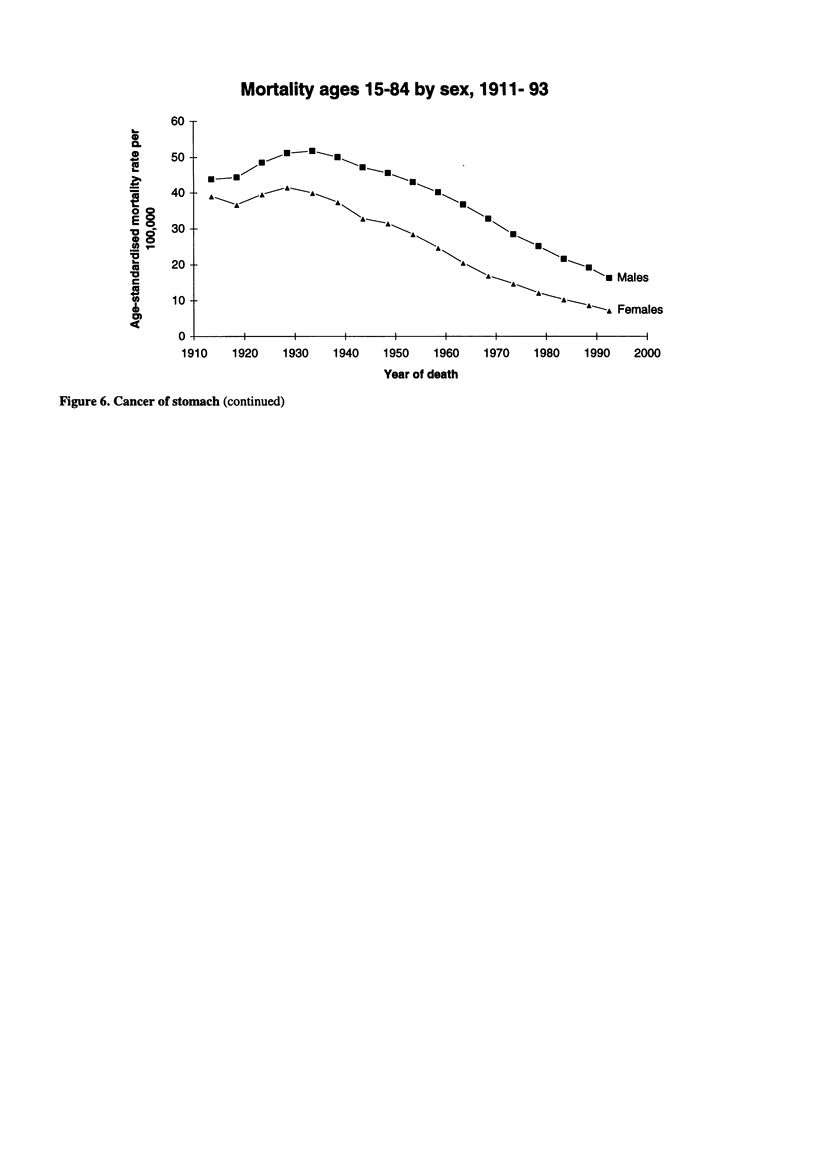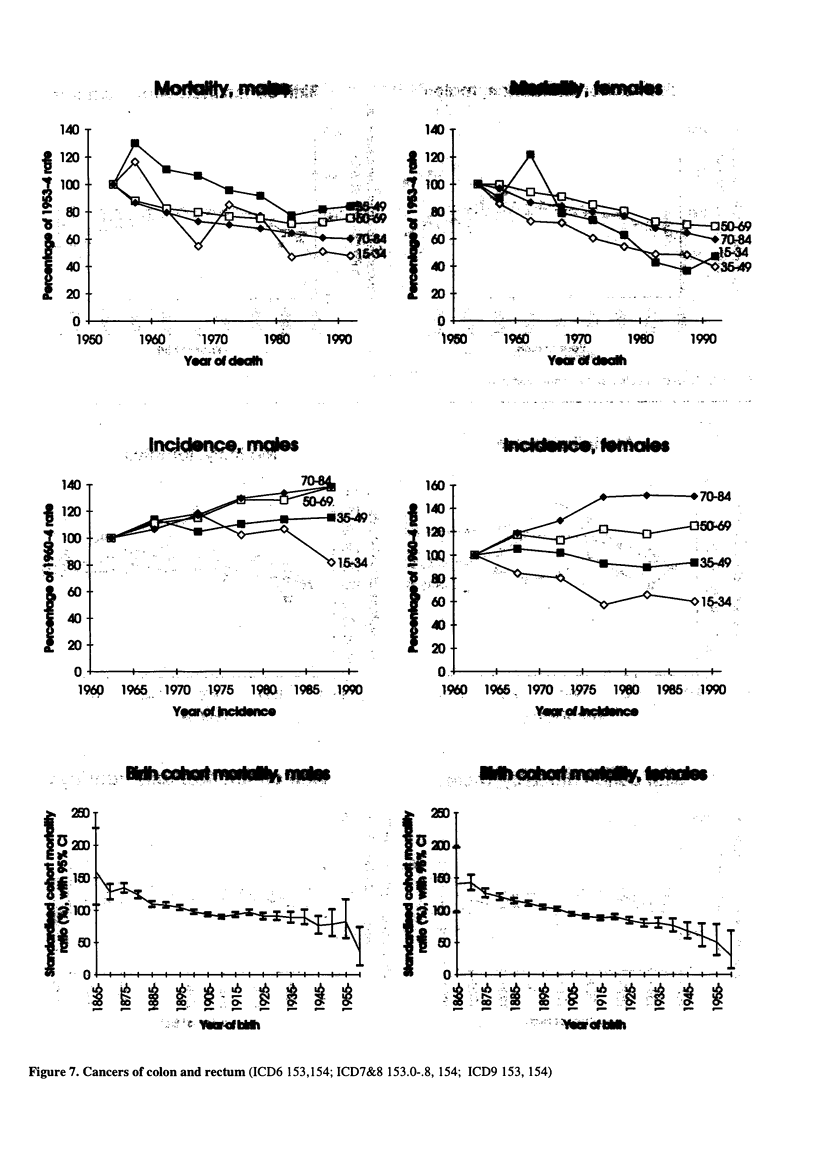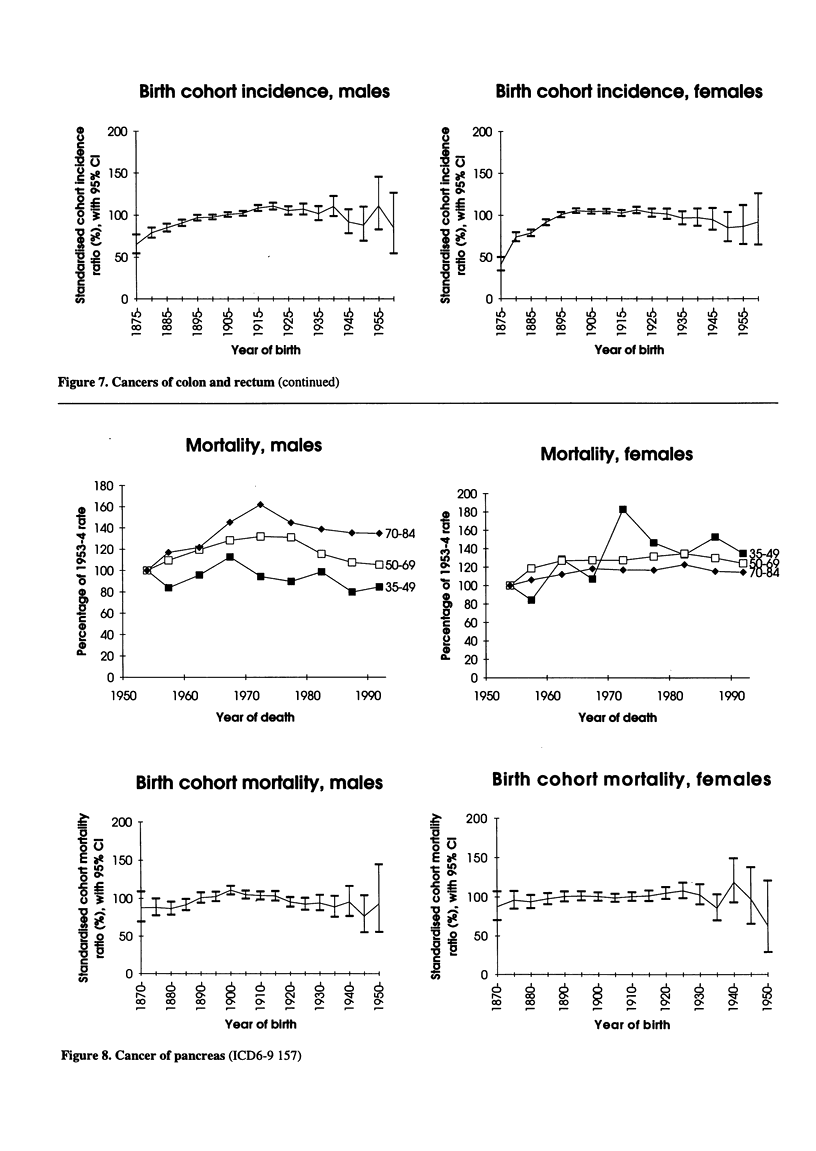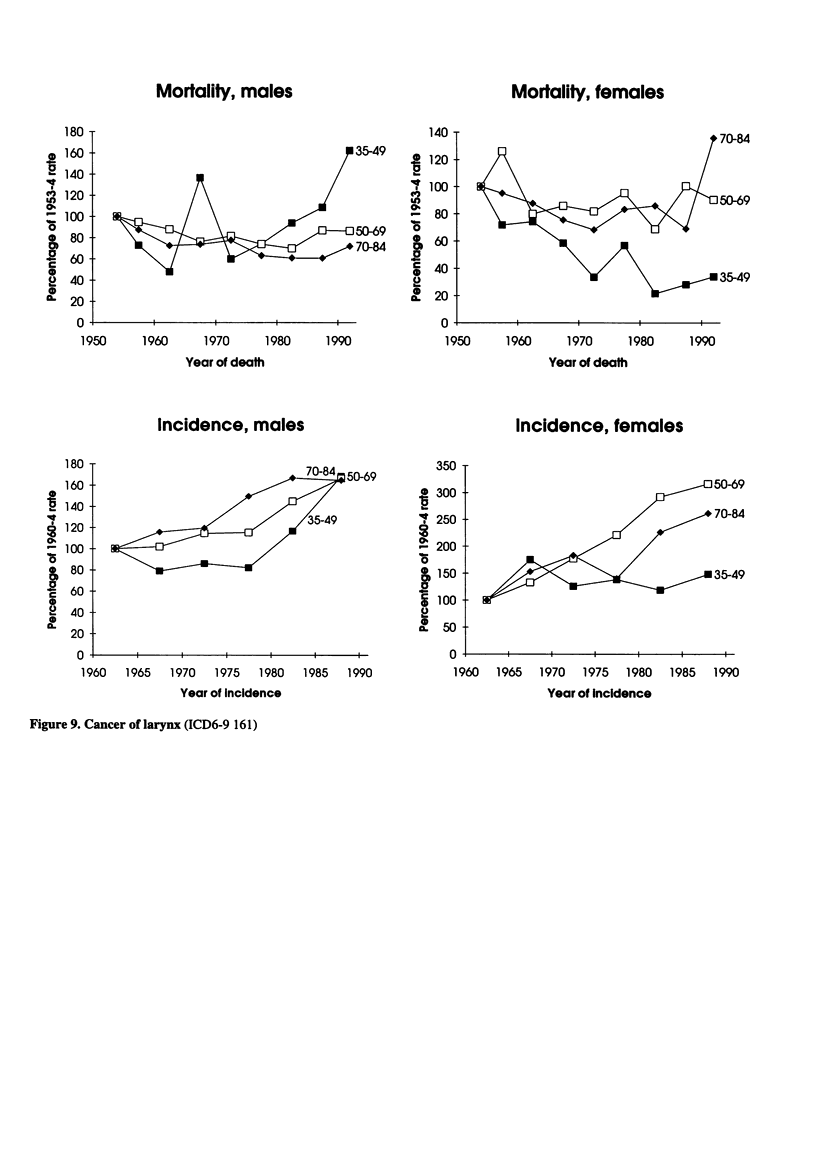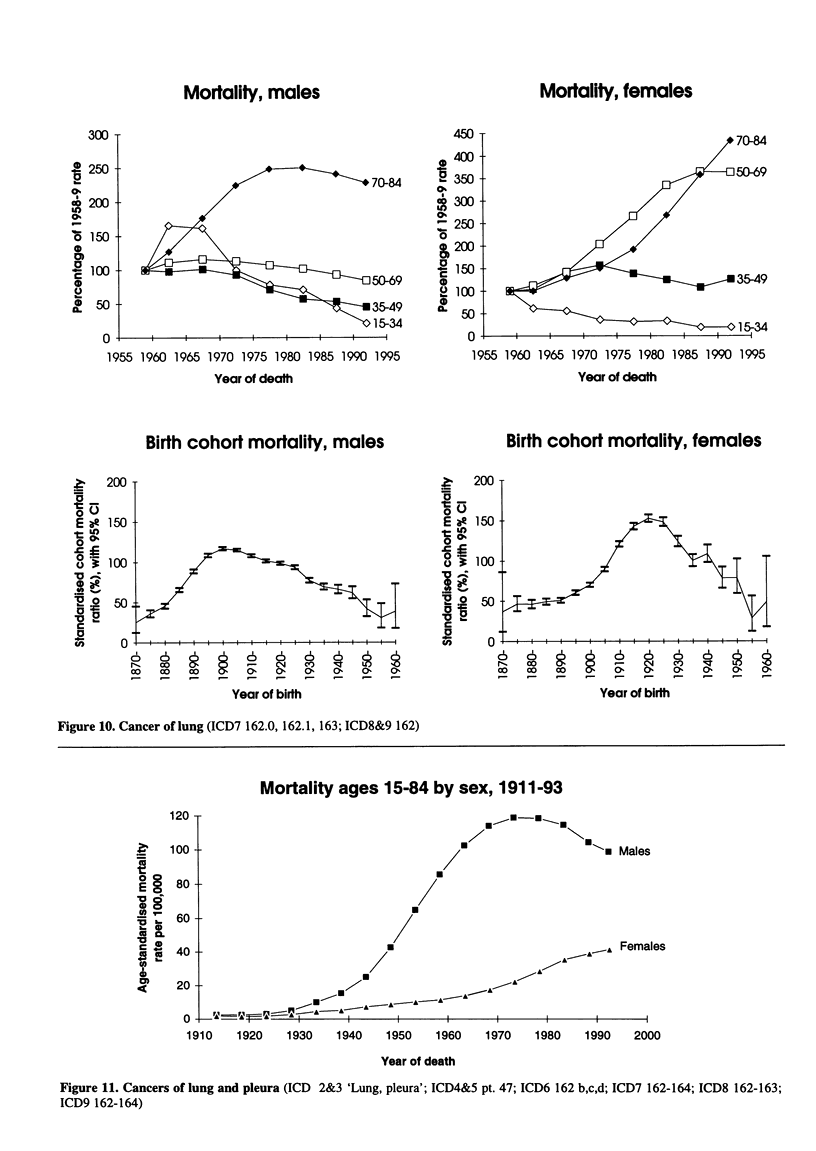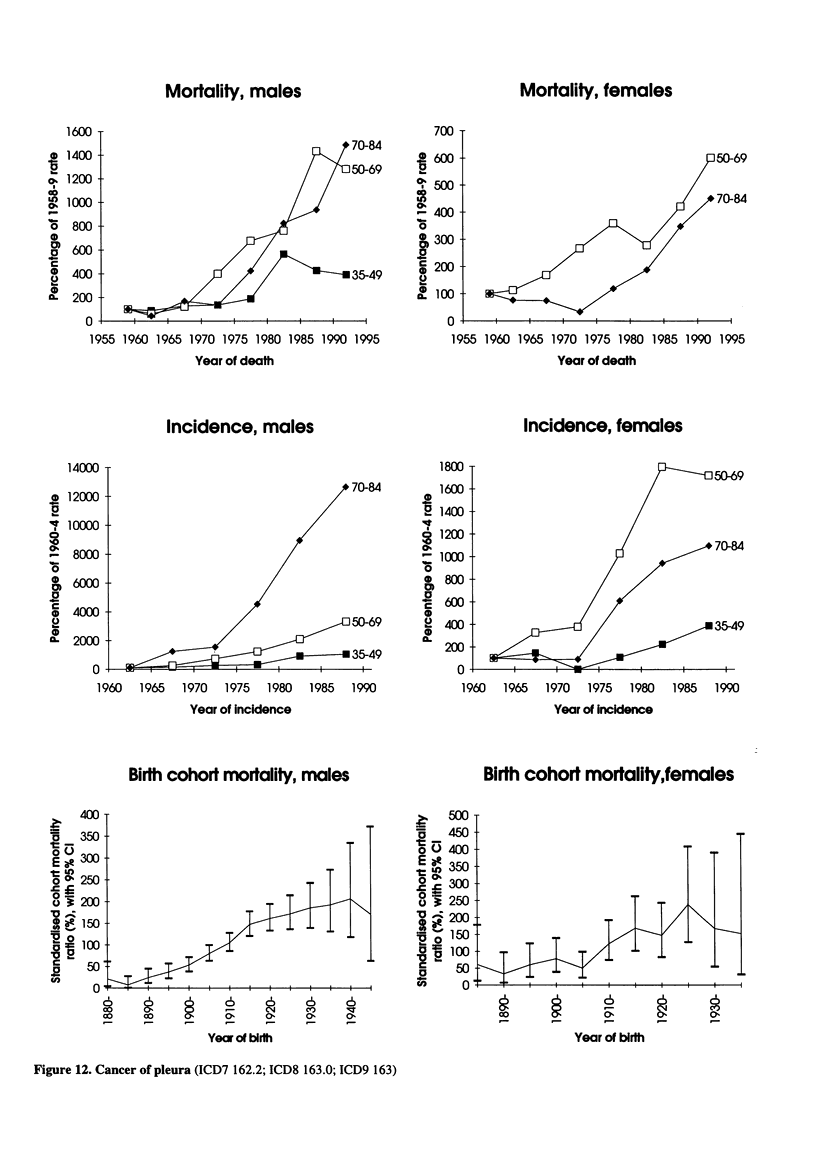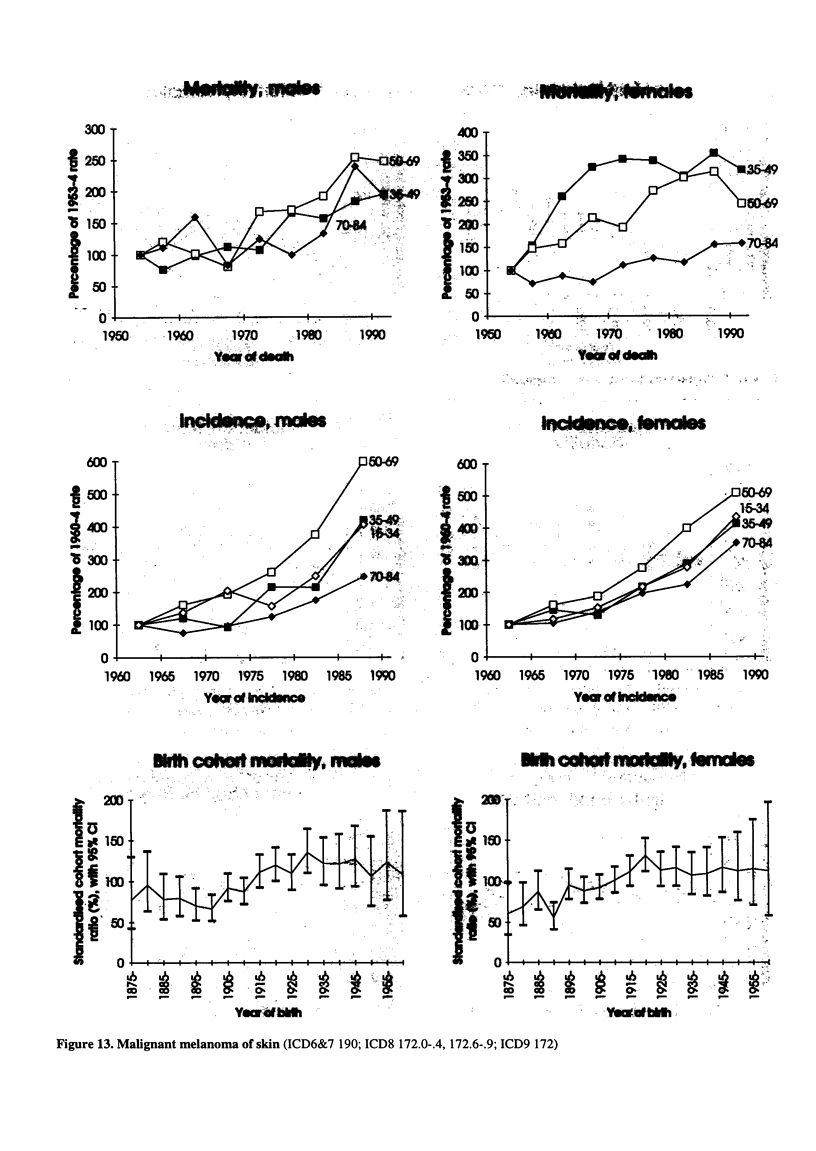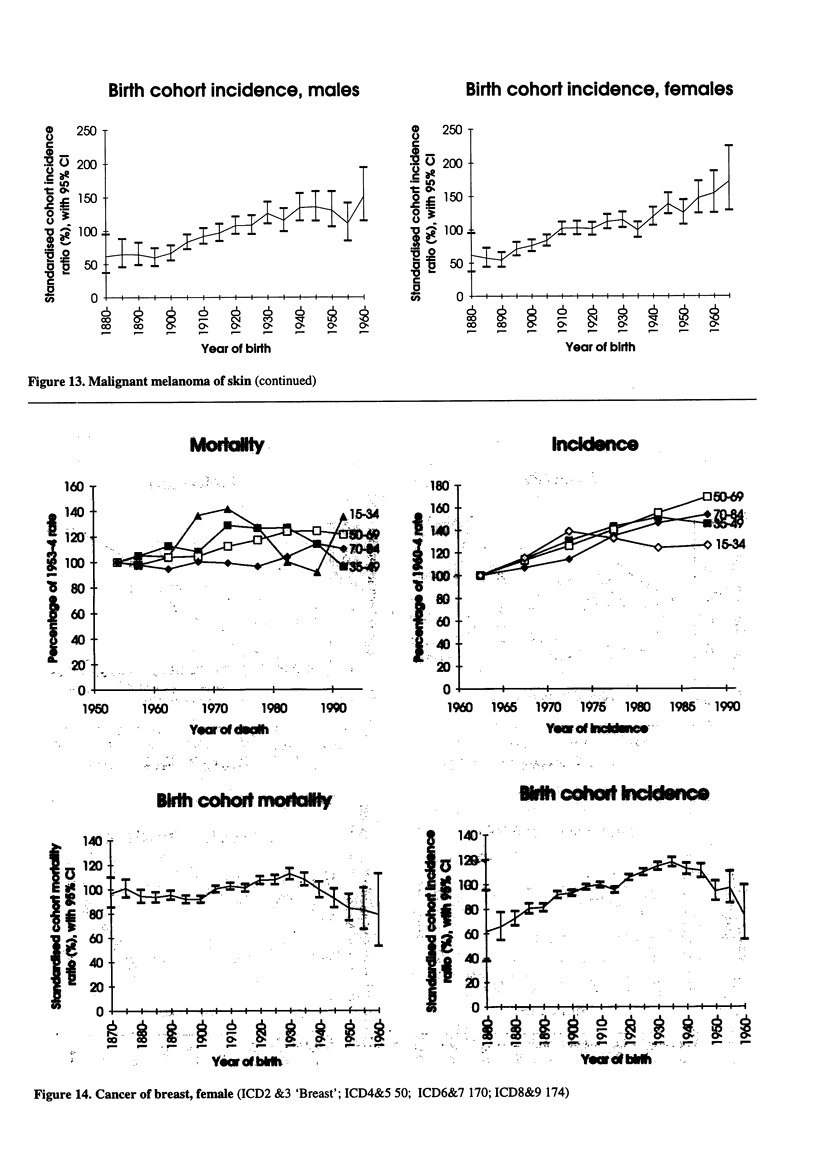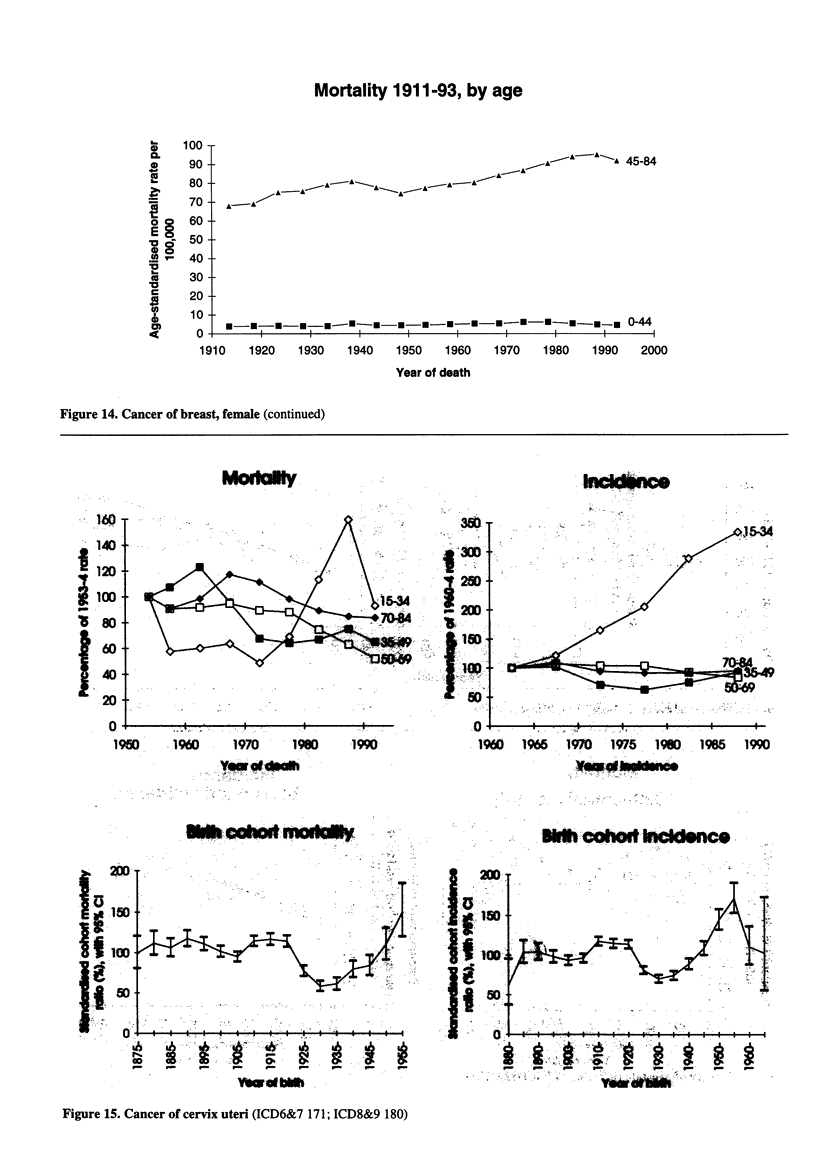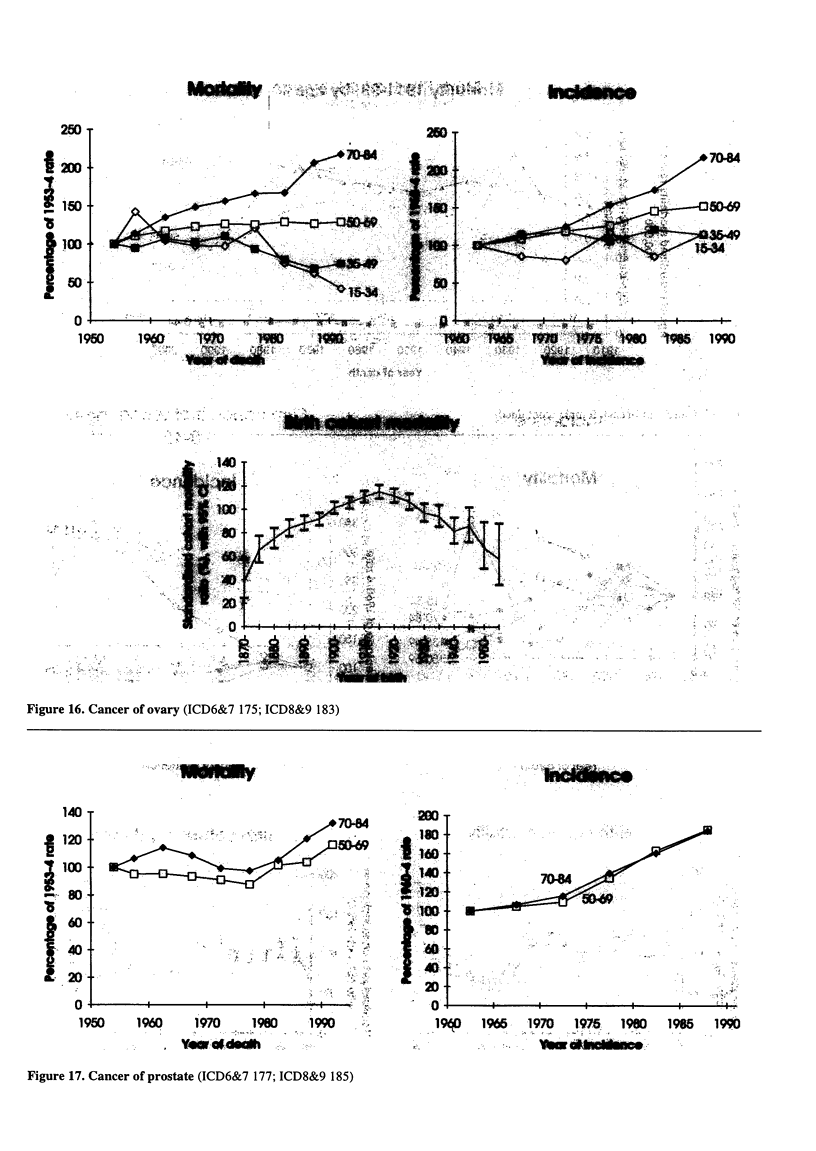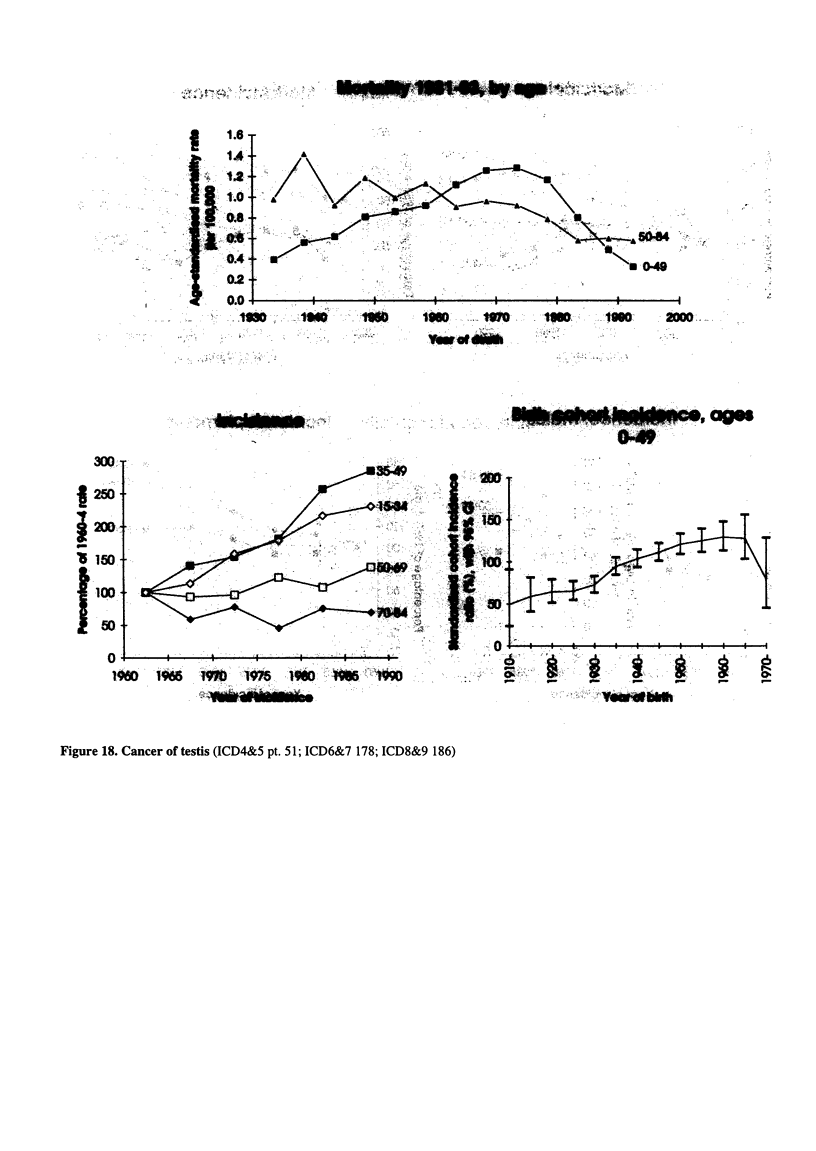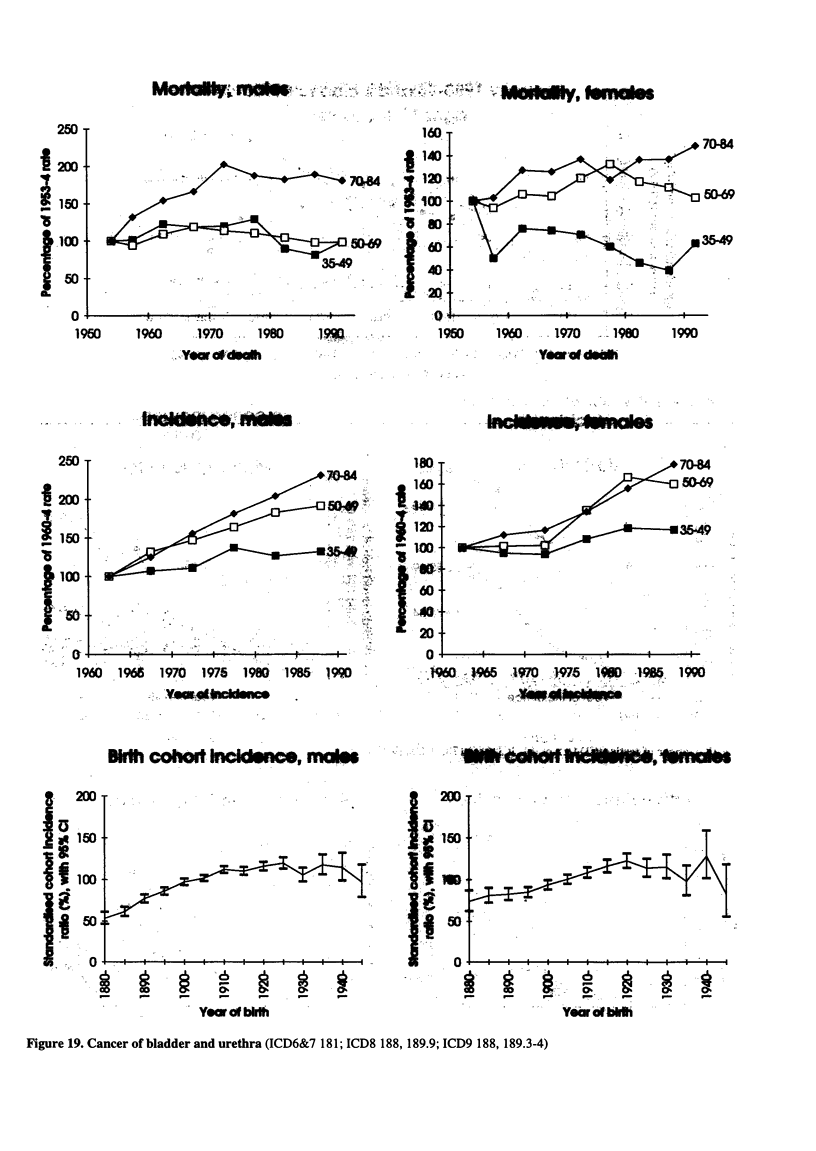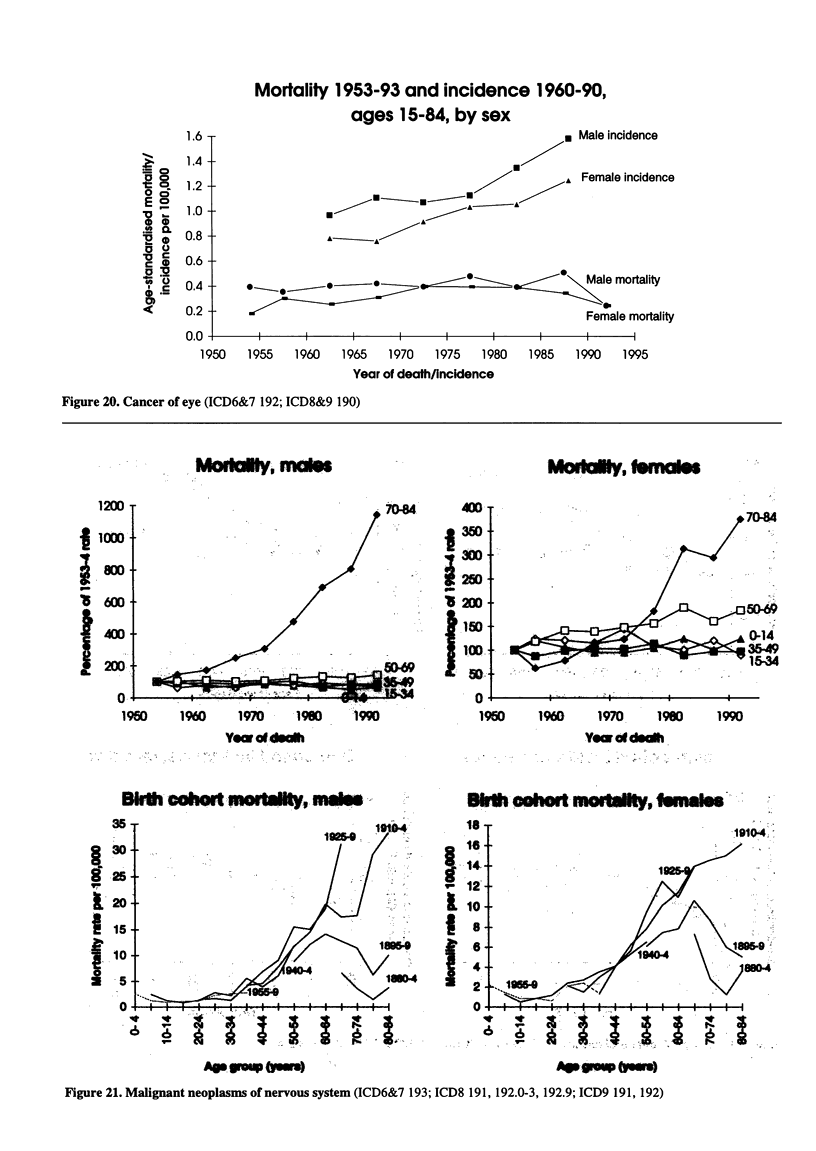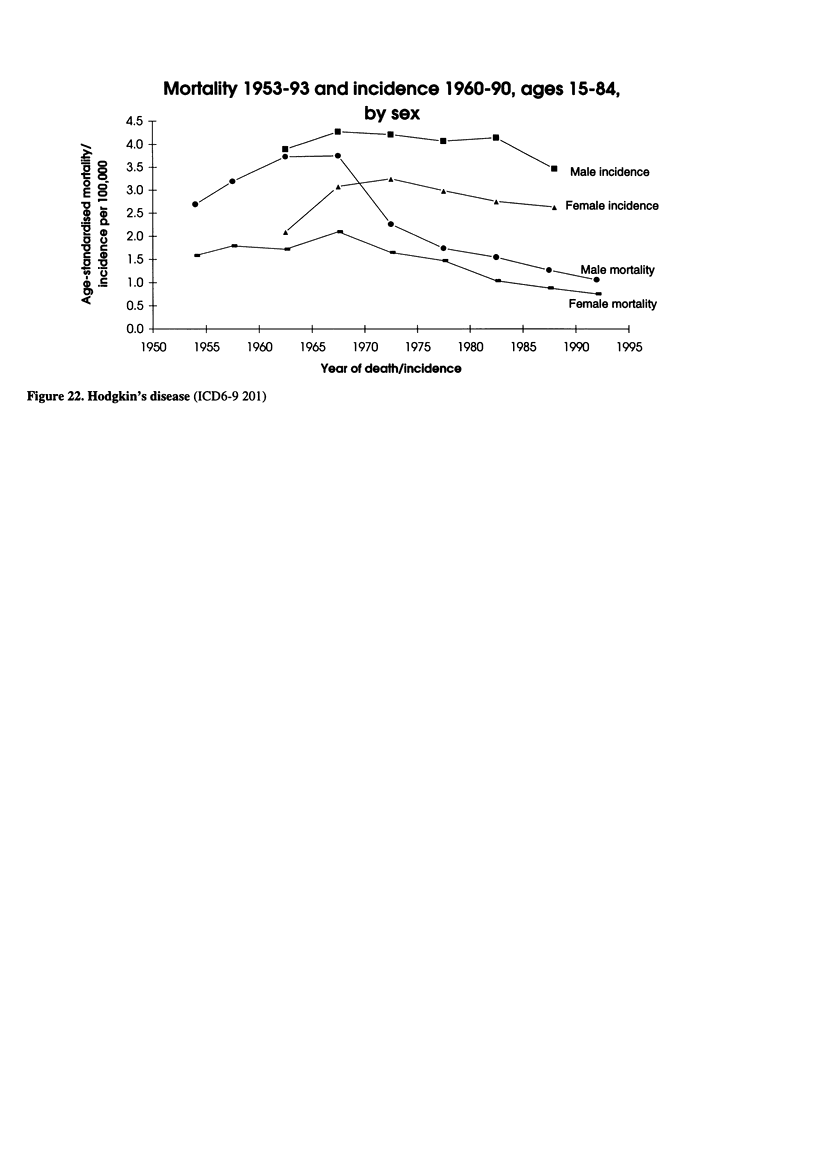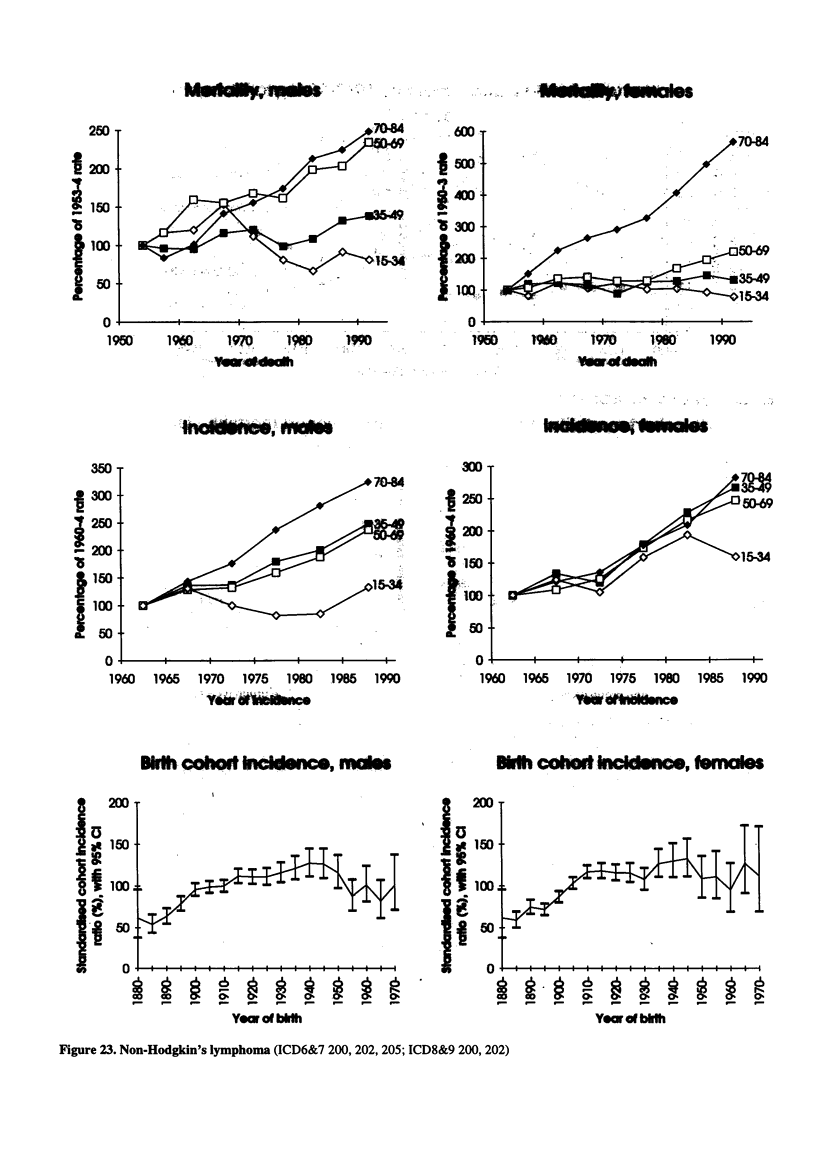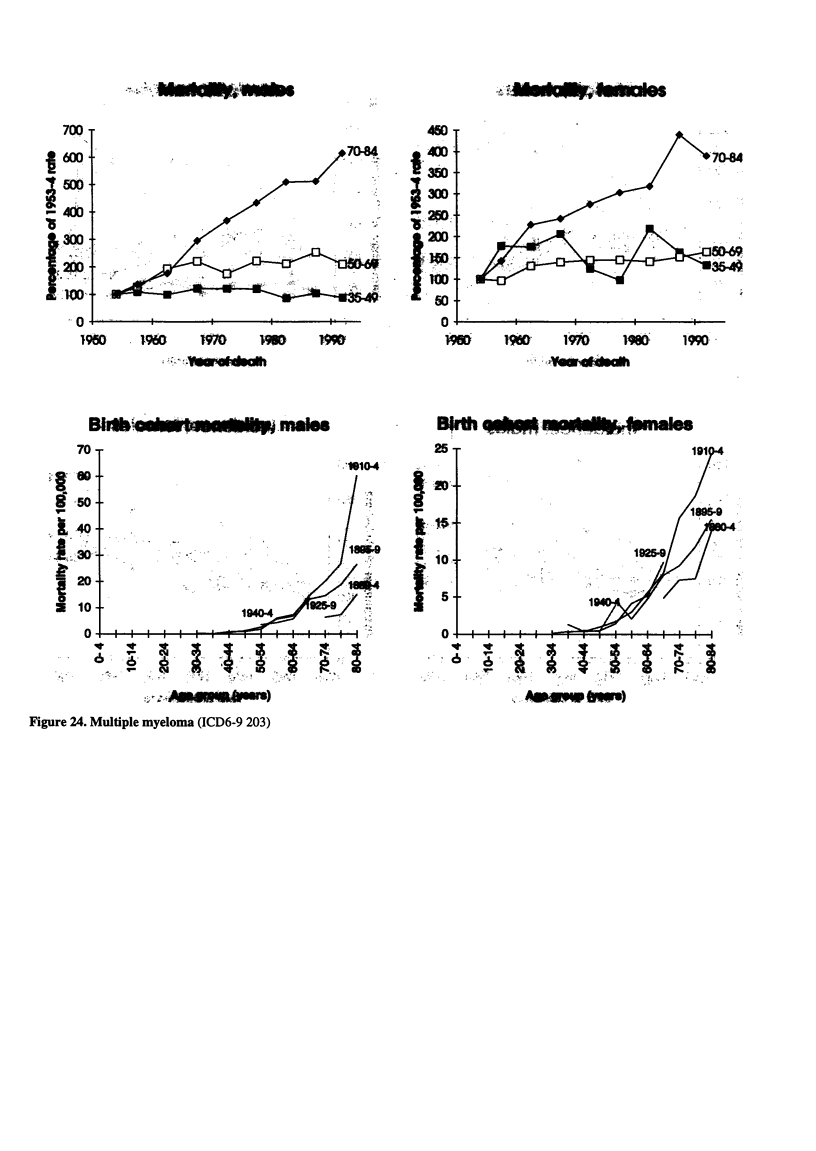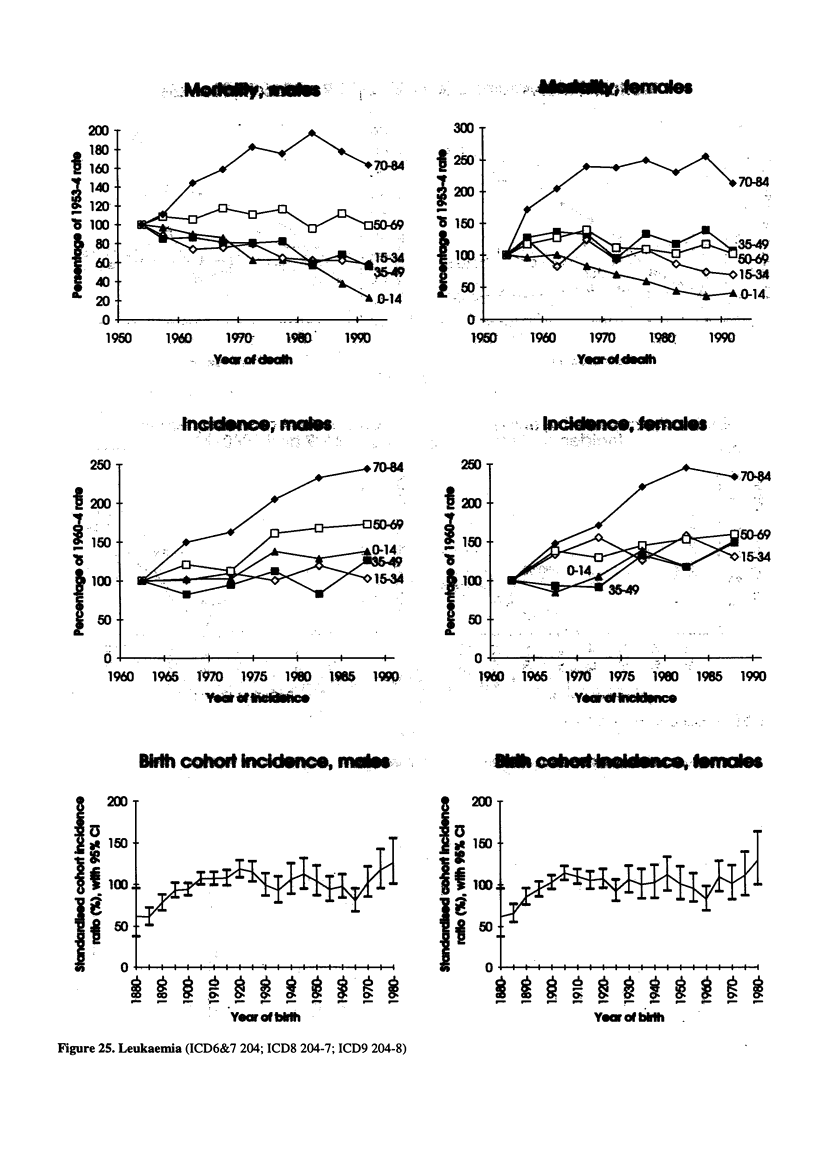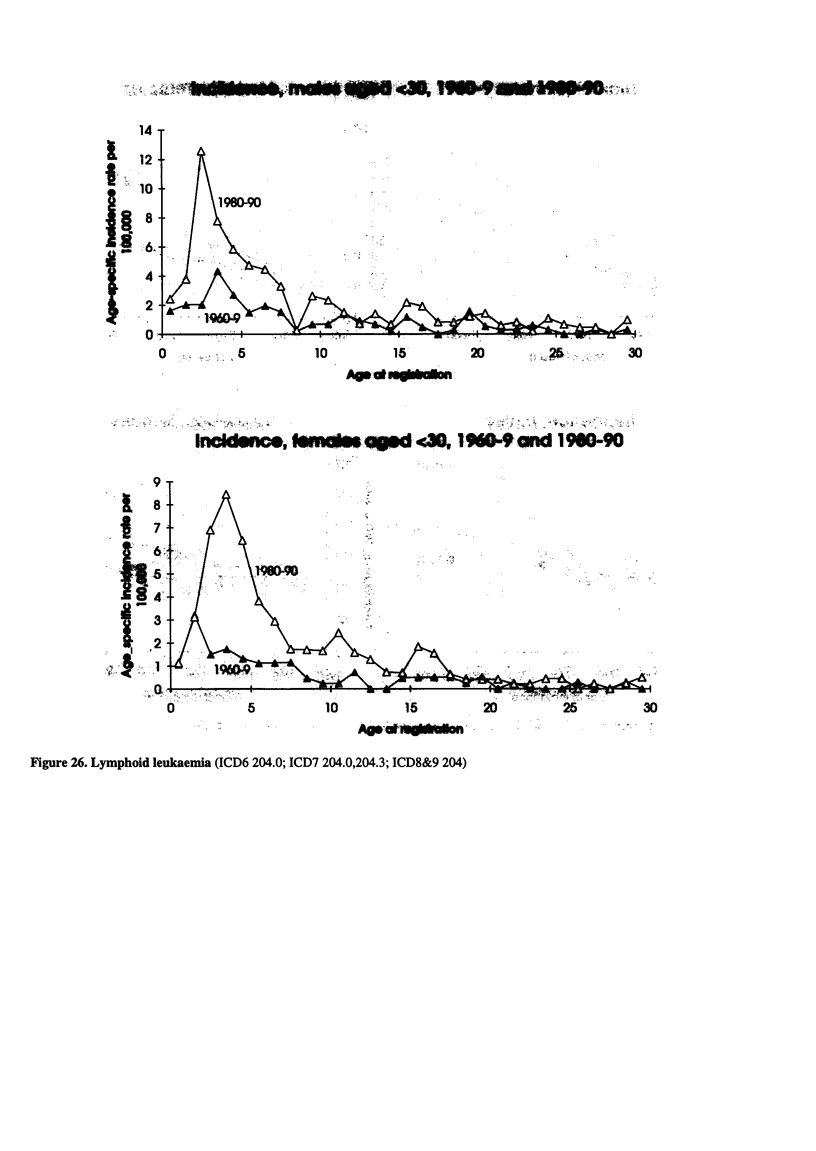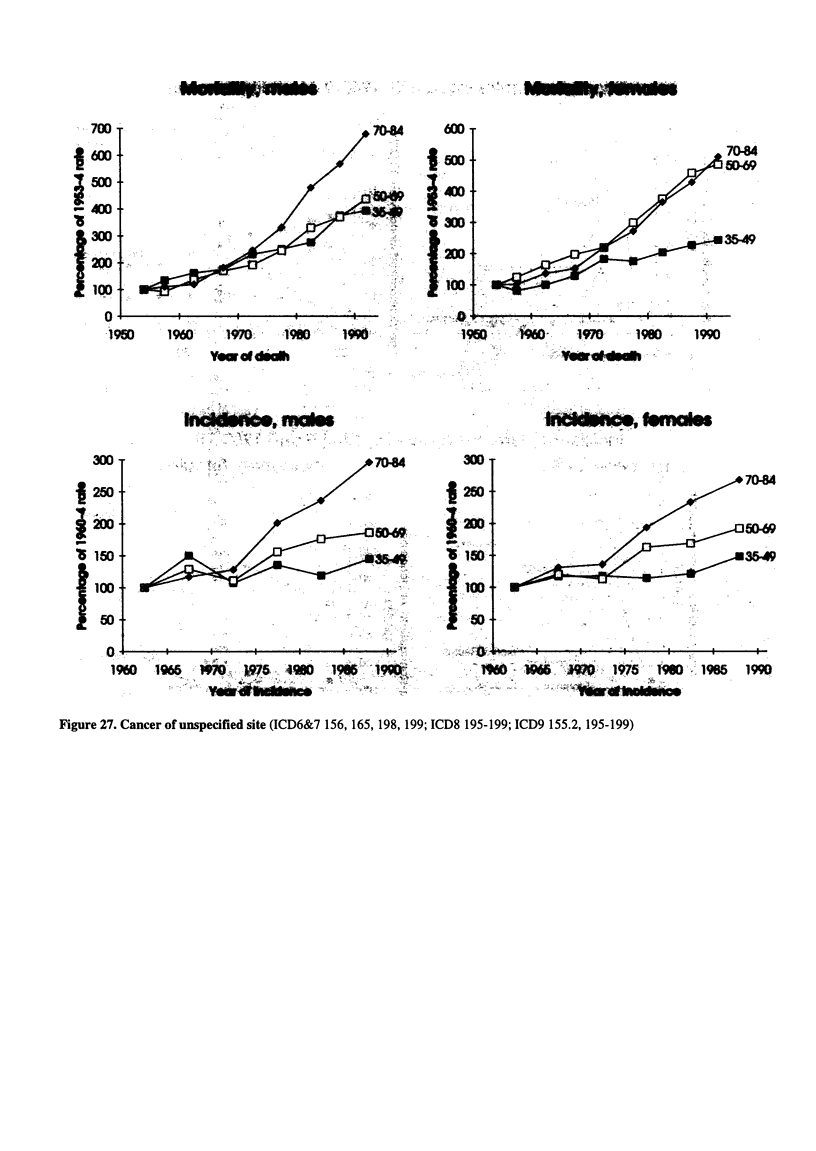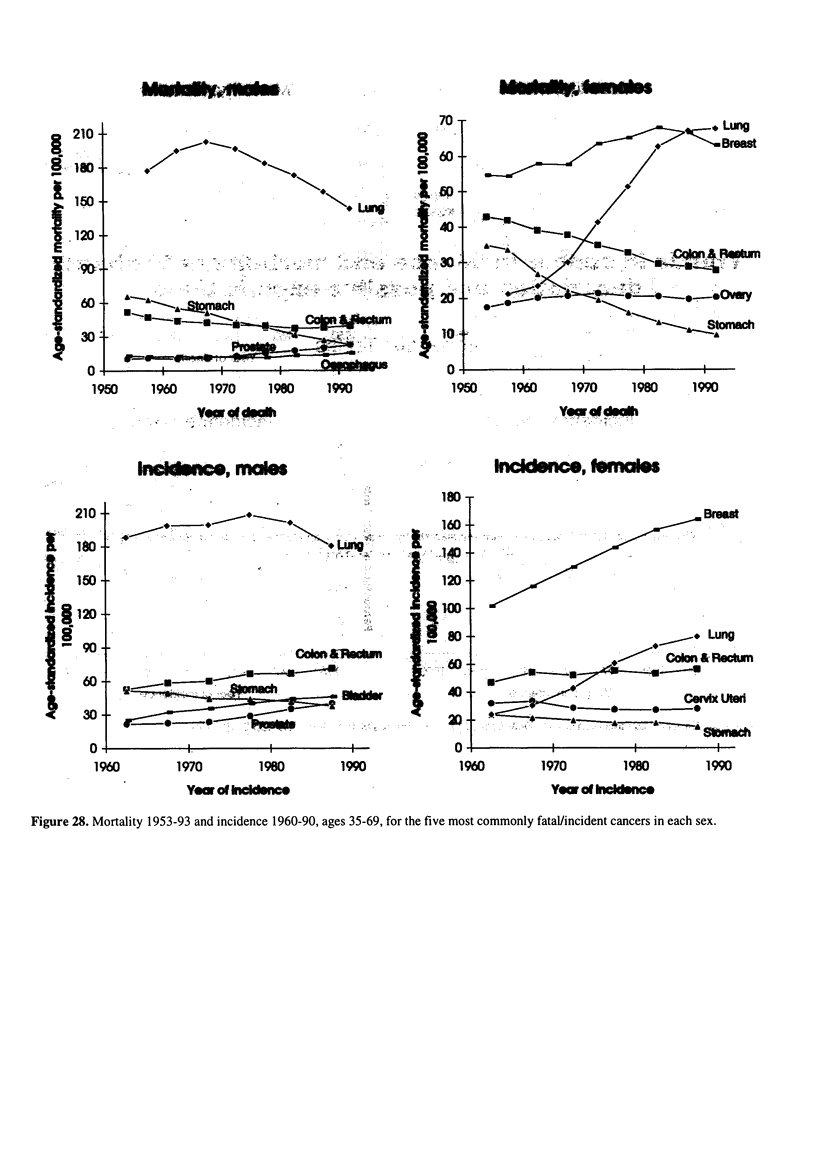# Trends in cancer incidence and mortality in Scotland: description and possible explanations

**Published:** 1998

**Authors:** 


					
British Joumal of Cancer (1998) 77(Supplement 3), 17-42
? 1998 Cancer Research Campaign

Trends in cancer incidence and mortality in Scotland:

description and possible explanations

Appendix: Figures

17

30'Death certificate only' casesmotly
30~~~~~~~~~~~~~~~~0

first accepted for registration

Z   25I/

~                            lDBrgisraton         IC920Hospital admission data  females, mortality
E                     ~~~~~~~first input to rei/rao

-0

50.             ICD7                                                          A~,Amales, incidence

A  ~ A  A  A~-A   ,-* females, incidence

co0    5                                        A4.~ ~ ~
0)

0)~~~~~~~~~~c

Year of deatM/ncidence

Figure 1. Mortality 1953-93 and incidence 1960-90 at ages 15-84 from cancer of unspecified site (ICD 6&7 156, 165, 198, 199;
ICD8 195-199; ICD9 155.2, 195-199)

'Death certificate only' cases
first accepted for registration

o  500                       10~~~~~ID8.           ICD9            AA~

T              ~~~~Hospital admission data                          males, incidence
.o 450                     first input to registration  \4/  '4'    A-A-A -A

400ICDA -/                                                                       V   les,incidence

I  I                 ~~~~~A-A-A' A  A"~

350 T         4

t  300 -j-        ~~A-A@=*A:-    .@             .males, mortality

E 3 250

-0 71,                                                        ~~~~~~~~~~~~~~~~~~~~~females, mortality
~ID6 200

150

150
00

0O

Year of deatMAncidence

Figure 2. Mortality 1953-93 and incidence 1960-90 at ages 15-84 from all malignancies except non-melanoma skin cancer (ICD
6 140-190, 192-205, 292.3, 294; ICD 7 140-190, 192-207, 292.3, 294; ICD8 140-172, 173.5, 174-209; ICD9 140-172, 174-208 and,
only available for mortality data, 238.4, 289.8)

RS,

-.:.:;... .....n sa...   .. . -... ..   :a

120
1 60

I:X

.0;

i                                                          I                             I i

lw9. . .  9.  198

VYof deah

1990

0-914

..  *.. -  I  .  . I  .  .  .. . .  _

195  0     . ..-  1970 . ... '18.0- I

V.wel de t

~~.        . .

I   .n c   s - n c ..  ..

.  qw W.   - ,...I  ..

.. :   . : ...   ..   ..   ..

180 -

160 .
140.-

120 - .

100 .U.

680

60   7 s....

:...

*40-_
20

0
1960

1965 ;  1970  1975  1980   19865  1990

Yen           . .        .cIdifl

Sm h  at      f nh

120

fl~ ~ ~    ~~Vra bblh ||||

80.~~~~~~~~~~~~~~~~~~~~~~~~~~~~~~~~~~~.:

r;w Y"d bbh

, ,. 160

1140
....

. -. .10   -

180

600

20 .0

ao..

U54 9

A:iXB:~~~~~~~01

..       I..       I *        - .  1    I.  . .  -  .  i

1960  1965  1970  1975  1900 1965  1990

YVm,. hd.s h .t.

.I

...

.I

I

.

...- =_ ss . .

Cw --

; .; .. ': .. .o. ,. , . : s . . : . b-X.:, .> :,. -_.;

. , .

-.0

e

a~~~~

V.   _  ..

Yodelhi

Figure 3. All malignancies except non-melanoma skin cancer (ICD6 140-190, 192-205, 292.3, 294; ICD7 140-190, 192-207, 292.3,
294; ICD8 140-172, 173.5, 174-209; ICD9 140-172, 174-208 and, only available for mortality data, 238.4, 289.8)

. I   .        . _..

I

'I

is

160 1.
140 -

120:.
100 --
80.
60 --
40 -
20 -.

0 -...

1950

1990

I

I

Ia

Birth cohort incidence, males

Birth cohort incidence, females

0

0    160

C

. c) 140

c a  120
s     100

0

?    80

a   6 60

, ?    40
.a 20
C

.2      0

(lb

o6       6 o        o   o  o  o  o

C00     O  -  C14 Yea) o bi rth

00 0       ol ol ol     C>  01 C> b

Year of birth

Figure 3. All malignancies except non-melanoma skin cancer (continued)

Mortality, males

140 T

.s 120-
a

'?1 00

80
ot 60
to

0)

er- 40-

0* 20 -

0 4

1950

1960       1970      1980       1990

Year of death

Birth cohort mortality, males

Mortality, females

i                                                     i                                                      i                                                     i

1960      1970      1980      1990

Year of death

Birth cohort mortality, females

.k   250

? U 200

E ae

c c 150
8 -g

a S 100

20

'o      0

o, -1.  6 9         A       4Ya o b 6i r

Year d birth

Figure 4. Cancers of tongue, mouth and pharynx (ICD6&7 141, 143-5, 147-8; ICD8&9 141, 143-6, 148-9)

0

o    160

c

0 o 140

120

0c 0 1 20

._

? ?   80

f -

** 0  40

c.. 20
C

.2     0
(lb

00   cf  o  -  cs-   cO sr0s

oo~~~~~. aa"   ""  aK oK  o,  o,  o

Year of birth

450 -
* 400-

. 350-
; 300-

_ 250-
"6

0 200 -
2 150-
0

2  100   -

0

0'50 -

0-
1950

2    300

1

* U 250
E e

to 1  200

0

o    150

_    1 00

2o

a a   50

.2

(0     0

O    0    0   O    O    O   O6   O

r    oo c o        -    CYe  of birt

co  co   co   c    ol  0ov  0.   ov   c,

Year of birth

i i 0 a i i i i a i m i i a i i i M i I

-1

-0

Mortality, males

.b2

2

01

0)

C.)

C
0

2
0

a.

1960      1970      1980     1990

Year of death

140 -
120 -
100 -
80 -
60-
40 -

20 U

9-5

1950

Mortality, females

1960     1970      1980     1990

Year of death

Birth cohort mortality,males

Birth cohort mortality,females

6    200

0

.CE

oi ~ 100
la ..Z

o  e

.2

U)      A

O  g     6 O   O   O   O           6  g

00  OC)  _ l O l b   a  o l b00    c0  00

Year of birth

Mortality ages 15-84 by sex, 1911-93

6 6
Year of birth

* males
*~~~~~~~~L_l ~~~~~~~~~~~I -"

*                                   A-A,--'A females

AA --

A---- ~ ~ ~ ~   ~   ~   ~   -

1910    1920    1930    1940    1950    1960

Year of Death

1970    1980    1990   2000

Figure 5. Cancer of oesophagus (ICD2&3 'Oesophagus'; ICD4&5 pt. 46; ICD6-9 150)

300 -
l 250-
L 200-

"6 150-
0
0)

0

0 50 -
O-

90
1950

.     200
r! (,

0

o z   100

o be

v     =  50

'O2
c
.2

U)       n)

U

6

co)
0o.

6

0

N-.
00

0

8

O0

0 O

* 0.

C...'

'.0

Ea

cm -.
<D .

16 -
14 -
12 -
10 -
8 -
6 -
4-
2-
0

i                                     i                                      i                                     i                                     i                                     i                                      i
i

uI

ML;.;, . 4     : *   * - c.;  _

120

'7

~80.

160.
140
120

0

19io    1960    1970-    1980    1990

* s ar: "d

3-a

120
100

6.

140

0--         I    I  -----  --I

1960 1965 1970 1975 198  198  199

Ye @1 tiSSues

flh   Y of moci   , mnc

200

U

O. 150

100

n

100 T

XI.- -  4
so*

: 2D -

.:  I -C  20u

n.4

1980

190- -  190    1'0

..  ... A - --   . I  -. .

* .---   1 d- W dh

1990

-~~~~~~- pP M a

100 T

I               I      I -          I  -

1960   1965  919     1975  1980   1985   1990

YeofI

Mh c

Ydb

Year o btm

Figure 6. Cancer of stomach (ICD2&3 'Stomach'; ICD4&5 pt. 46; 1

[CD6-9 151)

*a

-.   g     g1     -     22

_   _   _   _   _   _   _   _   _   _~~~a

so YdW

s

i            . -.-            .        i                                   i

so -

Om 60 -
Is

40 -

20"

I

u I

Mortality ages 15-84 by sex, 1911- 93

60 T

Males

Females

i                                               i                                                                                              i                                              i                                              i                                               i                                               i                                              I

1910    1920    1930    1940    1950    1960    1970   1980    1990    2000

Year of death

Figure 6. Cancer of stomach (continued)

Q
0.

0     50-

40

X 8   30-

.8

-0

<x,   20-

0

0     10-
Oa-

0  0

I-

I-

lkm-       11   ml                       .,.  - -

I I im      ?In--tt-
?,Www- ???"             WWI

vi -.-       Mlp?         - .;.

I ?'.. . - 0.

.   .   . ~ ~ ~ .. .  . ,

1960      1970     1980

. i *   *   .   ..   .   ...

Ye d dWdh

:-  -.;-:

,  .                            I

1965    1970   .-1975   19W8   1965,

Yac

140

I120.

1100

40

.  2... ...- ...

1990

.

1960     1970      1

*lt e., .,  . :.  1 -  eg

yew-di

YenSial

1980

M fls  -i b e s

W. li

.1990

160 -
I 140-

.0-

160
41) -

20 --

Q r-
1960

.       .  ..   ..  .   .  *            .

1966., 1970 --..1975                 198.

Y.n__     _       . .     ..

.. '  t_

. - .i .7.   "

- 1

:. V .d

. . .. .

lb  -e n

Figure 7. Cancers of colon and rectum (ICD6 153,154; ICD7&8 153.0-.8, 154; ICD9 153, 154)

140

120-
100-
80 -
.60-
40-

I

'7

it

20 --

1950

1990

140

1120-
t 17

-is

40-

20-

0 4-
1960

>154.

*2

|5D.

1985- .1990

I  1   -   ,.   | |

,                          .                          .              .....       .              ..      .    .                 .        .

.. _...... ... .......   .   .-

i_ .   , I .. . . , .... .. , , . _  ii , . , _ = ..... .- .

70-%
SG-69.

16-34.x

0

a .

. I   . . . . .  .   .   . . .

..            :. tb   ...6

0%,   ? r-

00'.. --.. ON9
r- r-      r- r-

- %%W-d bM

Birth cohort incidence, males

0     200

0

be 150-

.-I

0 oX

C

0         0

:- rg   -u

. _
C

*   200
0
C

0

ola  150

I0

0 3 100
0 0

=005
C

.   .   .I     I     i   i   i   i   i  I  (a   C o o0 ~ c

00l.  00  00OO.C> C> C

0  cov co R Y -e  o  i  rt h

Year of birth

Birth cohort incidence, females

) - a-r~~~~~~~. it

rl-  a)  oK    o   -    04   co   v    u
00   00   00  C     .   (>   C>   a    C

Year of birth

Figure 7. Cancers of colon and rectum (continued)

200
* 180
, 70-84  2 160

* 140
50-69   0 120
135-49   o 100

&  80

c 60

0

2 40

0

1fi  20
-            0

1950      1960       1970      1980      1990

Year of death

Birth cohort mortality, males

Mortality, females

-.--=  -   _ 3-49.

i                i                 i                i

1950      1960     1970      1980      1990

Year of death

Birth cohort mortality, females

-2b  200

0

0-

E aR 150

ao   100

%.w

*2 o  50

Xc 2
C

(a  0

o   o    o           6    o

co  co  c0   o  C>   C>   c   C

Year of birth

Figure 8. Cancer of pancreas (ICD6-9 157)

Mortality, males

180 -
*, 160 -
2 140 -

(  120 -
0.

_ 100 -
o   80-
0)

-2  60-
c

0

2   40-
0

L 20-

O 0

200
oO

E 0 150

o ? 100

2 o 50

.c -S

0

,  _

6    6     6    6    6    6     6    6    O

r-,  co    >    0    -    0     e     v   s

00   co   co    C>   C>   C>   C>    C>   C>

Year of birth

i          i           i          i          i           i          i           0          A           i          i           i          i           i          a           i          i

i                                                                                            i                                             i

Mortality, males

Mortality, females

140-
I '1F;AQ

4-

2

In

0.

50-69       I

> 70-84     0

.Q

c
0
ID

1960      1970     1980     1990

Year of death

120
100

80
60

40-

20

1950

1960     1970     1980     1990

Year of death

Incidence, males

Incidence, females

0~

0%
a.

350 -
300 -
250 -
200 -
150 -

100 - 1
50 -

0--
1960

1965  1970  1975   1980  1985  1990

Year of Incidence

Figure 9. Cancer of larynx (ICD6-9 161)

0

2
cn

0
.2

CL

180 -
160 -
140 -
120 -

100 -

80 -
60 -
40 -
20 -

0--
1950

180 -
160 -
140 -
120 -

100 -

80 -
60 -
40 -
20 -

0-
1960

2

a

a.

1965   1970  1975   1980   1985  1990

Year of Incidence

Mortality, males

300
250
200
150
100
50

->' 15-34

0 ) I  i    i    i     i    i    i    i

1955 1960 1965 1970 1975 1980 1985 1990 1995

Year of death

Birth cohort mortality, males

.    200
PC a

E O 150

o i 100

8? I_o

0 _

- r   50

b
a

U)     0

o          6          o 6 6 6 6

co  00   00  o     l _ . cs    9   o, u o

Year of birth

Figure 10. Cancer of lung (ICD7 162.0, 162.1, 163; ICD8&9 162)

Mortality, females

450                                           70-84

50-69
2 350

300

250
0

2200

_

E at 150  -3

2 1004

0               050

15-34

0n     u ~      I   I   I    I

1955 1960 1965 1970 1975 1980 1985 1990 1995

Year of death

Birth cohort mortality, females

., 200

3

E ae 150
0

o   100

ae

0    50                       I

0l-  co  co  _  K   b b

00  00  ( Y o00fO bi Or  O

Year of birth

Mortality ages 15-84 by sex, 1911-93

120-

.i'   100 +                                                                    U- Males

80

E~~~~~~~~~~~

,-,    60

Xa                                                                            . Females

a' 40

20-

0                                   I       I       I        I       I       I

1910    1920     1930    1940    1950     1960    1970    1980    1990     2000

Year of death

Figure 11. Cancers of lung and pleura (ICD 2&3 'Lung, pleura'; ICD4&5 pt. 47; ICD6 162 b,c,d; ICD7 162-164; ICD8 162-163;
ICD9 162-164)

0

2

0'

601

06
0%

0
0)
a-

Mortality, males

1600

70-84
1400 --

1200 --                             /       50-69
1000-/
800 --
600 -

400 3- 35-49
200 -I

1955 1960 1965 1970 1975 1980 1985 1990 1995

Year of death

Mortality, females

700 T

.s 600-
2

0 500-

- 400-
C 300 -

0200
.2

er- 100 -

0

1955 1960 1965 1970 1975 1980 1985 1990 1995

Year of death

Incidence, males

Incidence, females

> 70-84

0

0
s
0

]50-69      2

0
a.
'35-49

1965   1970  1975   1980   1985  1990

Year of incidence

1800 -
1600 -
1400 -
1200 -
1000 -
800 -
600 -
400 -
200 -

0-

1960

Birth cohort mortality, males

1965   1970  1975   1980

Year of incidence

-2~400-

*5  350

o U 300-

E ae

0 g 250

o  200

hiso
0  ' 1

C02o

6       6

co      ol
co      co

6     8
Yecr of dbirh

6

CII)

8

500

o Q

tIo
00

82
O

Co

6        6       6
Year of birth

Figure 12. Cancer of pleura (ICD7 162.2; ICD8 163.0; ICD9 163)

0.

06
0
0
.2
0
a.

14000
g 12000
2

I 10000

0

b 8000

c   6000
.2
c

0 4000

0

a. 2000

?

1960v

1960

1985 1990

Birth cohort mortality,females

i                        i                         i                        i                        i                         i                        i

300 .

.250 *-.
I

f . 00.

5.m .

150 .

@50 -
a.4

1950.

i                                                                                I                                       I

1960 . .... 19970   .. i .

*.e. .. .  dash

1990

400
I 350

I 150

.. 100 . ..

SO ' . .

*...19_0

1980-  1970 t 190  .1990
* Y.- f dsc

..         - ...

600                             56-69
600

p400

1200

1100

-1960 1966  1970 1975  1980 1985 1990

Ye. of *I.clbnco  .

ES cSfwfy,ms

600

600. - -

1960   1965   1970   1975  1980    1985   1990

Yewtlncldsnce

E oSw n.S, bxi

.6.6mb      .6   6.       '6@.6 ..#

Yiaotb   h                                U

Figure 13. Malignant melanoma of skin (ICD6&7 190; ICD8 172.0-.4, 172.6-.9; ICD9 172)

lb  _ lb _ _

.  . _.

:   :01

.        .. . .   .--                                s

I

-, .1. 1- 11

.....0011.             -a

!                     0     1

. .  U.,W'W'                  id   "

Birth cohort incidence, males

Birth cohort incidence, females

*    250

0

c

S 0 200

be
to'

o    150

50

o1

ao' 100

e

._

0       0

, :0  50
c
D9

On

Year of birth

co  co  _ >  9   a  o b b

Year of birth

Figure 13. Malignant melanoma of skin (continued)

I nc1 i n c e

180'

1I-s

- . e                       ?m

I.    _ ._. '.. :. '  .I

1960  1970   1980

Ye" dealSh

Birthcohort -

1990

14

pt

LA

-'   312 |:;-                    -        --  -  -

120

40a

*      l20      I       -                  I2

1960  1966  1970  1976  1980  1986  1990

Ye        > o-baw

U   S.   c h ort

Mt      -  -  3l ~~~~~~~~~~40+

,~~~~~~~~~~~~~~~~~6 -

_ _  _ _ _ _ _    i iX  _ _ _ _ _ _ _ _ _ _ __l' '  '' *   i' + >   @   ;
6;t    dt-            -   d l

Y e  of birli  ~~~~~~~yew cf1b iMt

Figure 14. Cancer of breast, female (ICD2 &3 'Breast'; ICD4&5 50; ICD6&7 170; ICD8&9 174)

*    250

0

: ? 200

c ae
-c n

U,

o :E 150

=

o ?:

._.

0 0   50

c
D2

O4

50

160
1400

loo.

1. : 20--.

0- 0

19

$

I,

0  I

s I kily -

Mortality 191 1-93, by age

00 -

90A .~^ A                                                              45-84
80            A.A
70   -    A. ./
60
50
40
30
20

10-

* ^ * * * *- *-* * *-*-* 0-44
0         I       I       I       I       I

1910    1920    1930    1940   1950    1960    1970    1980    1990   2000

Year of death

Figure 14. Cancer of breast, female (continued)

a

..  .                                                                                                      i                                         .        i

-196    1970    1980

yw- .sea

*1990

-A     _ _ _ .   I  .  di..

_._        ,_     r.lll

.   .    .   -.-   -  . .-

S                     ~~~~~~~~~~~~15-34
5300

*10

.A30   I.        .   I P /

19W  1986 1970 1975 1980 1965 1990
.immf'.;. n'

Mtcohoffc..,

. .-O

0. .150
w.a

.  t.  . I

|  .  -.

i  i     ;..   .      .        . i  I  i.w "   0  . 1  . ...'..          i     i     11

1i  o  ,               ..-i  | liiP

_*i  t  m..ef2SS.   j

Figure 15. Cancer of cervix uteri (ICD6&7 171; ICD8&9 180)

2- 11
0.
0

ad

o     I
E8

:0

0

ca

0)
.4

* 140

120 .

1100      I

0W-

160 -
. 40 .

.20     .

i. .0

19'    .

..  J.   . b7. ..MY.   .  - . ...

m  o | t. o .c:- ..w. #

4.

.

u

.     ~.  i~ . ' e4-/ 'Y  .2 ;i < ) S  q > V   $ "L

-' fF '.'t

%!s . . . ! t

- . . . .. . ,. .. ' , . ...

_    _    . ]  .   .       -    ;          -

F -^-- ' - EV?, . ' t  R - - ' o' *;  .: .  '  sx

i T., ...... ; . ,,,. . :

..... _.t, ... t, i./j.'i' . . ' f

196      [       :-sS-T' If

Figure 16. Cancer of ovary (ICD6&7 175; ICD8&9 183)

140
120

100 +

OD+

60
.20

0

1950  1960  1970

*   :   ~~.  : f

.W lI:d

_/                                    . r              .

_e                         .. . _ . .        . i

_E o . 'S.

. .

. . t.
r E ..

?S
-  .   ;   ,.  .  '  X

*     ,W       ,,

*               .^t   -'-:,

. ....

- , t

* ' s l.-;
.!s  .                      s.  .

,;         .r    ;      i     # '      ^'^    s

/      s   |            .-  .;  .

. Zl ^ . '; .

i ' 1; Z

. . ...

|      ._ 0souusW__oC l.   - -.l  ,,     V.

1980 i990 ,;:

... e. _,, ,, ,, . t , _ . :*

i_                            . .

__w

200

0                 .             1

196)   1      1970    1975   1980

;~~~~~~~bm ... .. i_

Figure 17. Cancer of prostate (ICD6&7 177; ICD8&9 185)

250

.,s .

la9

150.

*I.

+

0$-

1950

WS 10..0

,a.

1990

I

I

I-   -1
I5

19 t .1 ,9

198  199.

. -                  ..l         ..     -     . .  _       _ .-   ..               m   .

i                             s

ml.

i                                   !       .

3.                             70-84

t?.- I. .

W-69

.0"             .-.N    : ....  . ... .b.I

'r                        .  I
--I        -, i         iih

M
.. A

. ! ".-,-': M-0118- -l""m

. . -- 4r

..  "? . - .? -, , .. .. . - -m

I    ....  ..  , "   ,,  PI       :.  . .

.1 Ii,.h                     VW.
.. .      .  ....-Clo, I         ..!?!

* t:

.. i^:

. @:

* :l -B:.

* : .B.-

fj o;

: .

.: : :. a ,- .

. i ss b.

. '.:' . . .

l

:* v.o  .      .

4  .   -~~~~~~~~~~~~~~~~~

.  .  .:

.*             X .s2.sb.w...... OUC,4l<. ".

*0s' '' ~*04

Aw   o   * '  - .w-

ISO  IWO:  tOY  ..160  . 1.  . . 19.0::. 2000A..

*vanS   . . ... .

F;, ...  # . *. .|

.. _ . _   _     ....YC I b M h ...................

Figure 18. Cancer of testis (ICD4&5 pt. 51; ICD6&7 178; ICD8&9 186)

300:.

I 260

't 1,5.

. .100

.150

1 ....

z                    i   : :   : - .     ' -s : - : -   :-- -s -a s- -- -- P----P------ -----z             -

x w x . B - * w w x s

....      .        .....  .:.  .  .       ....   .           ..     -         S      .              .:

1SB T95'5 }. F. . -.. . ' ''-.'

- . . :

A i t _ i i *

. . ..

l

.* <  t.:  .. ,( ,  - m1
..       16O,~~1~ T   -

p70-84
3 50-9
,3549

1960   .1970        18        1.99                1960      1960      1970     1980      1990

.:.     ds -alh                                             You      dmd0

. .  ,   .   . :

_       ~  ~~~~ ..  . . .

I n c U f l~~~~~~- 1 f l t*I I

i 1970 1975 1980

Y. nO  d sa

*198

. .. ,' ,_

. 1

*         .  __   '  '.

LI.     199D        -

160-
10

...7.;   ;

.

.lao.

...   70-84

50-69

. 36'49

'  M                      '

1W.- 1 190: .196 l-  18 6- 1990

- . .d --..

':. S'

-U ..

*.. . 0

. .!

It:

i    ii           i  i   i  i         i.

t       _~~~~~,

..Yewof bldh

Go      .     .. ON

r- ~ ~d r-  m l"  r    i

Figure 19. Cancer of bladder and urethra (ICD6&7 181; ICD8 188, 189.9; ICD9 188, 189.3-4)

260

11

120

is

'lao

jao

O 4-
1960

250 T

.

+

1.i200

, .--..

160.

|1

-. -     1

960  196i

S..

10

'a'

200 T

160

100+

S0
0 0

.  . .  , a  a.  . . .

-

nv

1 V-ww

. 0
k

L

.   .:.     .                                              7: ,

1.4 -
1.2 -
1.0 A
0.8 -
0.6 -
0.4 -
0.2 -
0.0 -

Mortality 1953-93 and incidence 1960-90,

ages 15-84, by sex

* Male incidence

A Female incidence

*   Male mortality

Female mortality

i-                                                   i                                                    i                                                     i                                                   i                                                    i                                                    i                                                    i                                                    i                                                     I

1950   1955    1960   1965   1970   1975   1980

Year of death/incidence

1985    1990   1995

Figure 20. Cancer of eye (ICD6&7 192; ICD8&9 190)

M, moWs

M w. h I maWs

1200

00 .
i 8600 -

p400 -

. .   I

02oI

1960

1960    1970     190

Yew of de 6h

199

35
30
.25
20

10

940 4

0

.   ,.,6

400- -
360

30 -s -
200

JiOD.,. .

1 90S. .

,950

70-84

360-9

0-14

I3&49

1&34

19603    1970     1960

Yew of dodlN

1990

-t _? J@|wlal|s

16~~~~~~~~~~~~~9
12-

9 190-4

0    _ 4   _4- - I i -  _   .   .. 4.s.------ - ............   - -

|10.4

.9

060-

1880 4

A    .p VOW .ws)                                           'v aV    vee)

Figure 21. Malignant neoplasms of nervous system (ICD6&7 193; ICD8 191, 192.0-3, 192.9; ICD9 191, 192)

1.6 -

t 0
o 0

Od
E 0
'O ..
0 0

.O r-

C.

I

f

i~~~~ _. _s_ .  -  7.s

V       V

v-                             11

46  -   6       46,.                   6

r- .-

. q

-t

Mortality 1953-93 and incidence 1960-90, ages 15-84,
4.5                                     by sex

%%      4.0

e0     o 35+                                                                   *  Male incidence

16 3.0                                                           -------

]  !    2.5 +                         /                                           Female inciden

2.0

Xc C                     _   _        _       _        _       _
C       1.5

1.0 +*----Male mortalil
0)    1.

<   0.5 - -                                                                Female mortali

0 .0         I        I        I        I        I        I        I        I        I

1950     1955     1960     1965     1970     1975     1980     1985     1990     1995

Year of death/incidence
Figure 22. Hodgkin's disease (ICD6-9 201)

ice

.._     ; _6- a n

* I     1  .  I

1. 170  1l0 199

..:V .-..I-de

.600.

5 Wa
|  400w

.0

1960D   190

.:

1970   190

i

_wn-

5 300

1150 -
. 1.

*.i.m'

0 '

350

7-84

3003

1960     1965    1970     1975    1980     1985    1990

*. e'&a

b cohdL

8200                                   2

IsSO                                    15
t100                                     0

j i s o                                  s o___ _  1

_ __ __ __ ___                      5

Yew d bidN

Figure 23. Non-Hodgkin's lymphoma (ICD6&7 200, 202, 205; ICD8&9 200, 202)

QO-84
'35-9

1960    1965     1970    1975    1980     1985    1990

... , s   arn.

_-Vb im.

1Mb coodio  cmc Imis

i'6             6

_  _      _ _ _

O.,  d  bE

250 -
200

9 1SO0 .
'V

|11:

0 -

1950'

1990

l a..s .

s        i  - -   -- -   l -

o
n .

-,;  0  1. -.1, I

Aftis .r ?

- - 9

mutes

700
j600
4500

3 00

198

0l  .     !            v

196    1    .X.90  90 1

Blat             mob

70 -

* ..

.a..'' ..

850.

is

g 20=-
2 10-

n

19404
;.F.

i          i          i           i           i          i                      I

_i. u. 24   .4.  .  .   .203.
Figure 24. Multiple myeloma (ICD6-9 203)

450

9 M... .

I an
e. TO

60

of 3 r

lowP

I  I. . .   -

1930    1WO     1990

in  :ldh

*310.4

(1u04    '

#'

{.1? ?.V

..       t '.

. '

ct 'ct      r

-l-                - 6

*.. .  .   .  -:  I  "! -.- -  ..I

;. AN   ..  r n)

1990

25; T                                                         11

1 'I -,

u -4

200
180

1160

140
120

'8100

.60:..

- 0        I .       - .  -I .. -  .I.  .  .  '  I

1950   1.960?  1970  - .   1990

-w -ofdh

260 -

200 -.

p-160
'8

jioo

300

25'.
ql

5 200

l 100. '- , ;

-   6 -.0

19501

260 T

-
I' :

*   r  0

1   '   '  -  - I  - M ..  j  .  I   i   I       .

1960    1965    1970   1976    1       1985   19w)

Y    wt          . . M I_

BMh cSdh  , -_

3  200

O

I100 ~

-, r . .

1960    197D     198Q

Ywot*alh

1990

o- -
' 1960

..  . . .

I _

ii

.,- i ...    -. . _  _

-.. .   I.

1965    1970   1975    1980   1986

....  ...   Ick  n

-  _ W .    _ w

.-- s

vi- p-  -  ' -  v- 0-- r- o.      P-

-  O Yofdm

Figure 25. Leukaemia (ICD6&7 204; ICD8 204-7; ICD9 204-8)

1990

Yr of blh =

a-

I -
- It             W-- W

:.  .:  . .  .  .  .  "%. ...  '.d

-L 1 -Ed.

.?  I.-   ,                            I         I                        .  .   . I

.... e                        I - - . -  V   ?- -14-. T .-                     .

.      1.  c   .  " .1 .  :.. :!   ....  ..

.   .  i

--m--

-1 I m.- P,---

-d       "' I- Ift is

:'' ! .'- :  I   -dil-  W   --. -  -- -

14

I    12 -

110               198D-90

It

0          I  1    0.  .  .   .  .   .

O , 1.  5  1 0  1D S: - ; .   ..   3

I1.Sc, _: 4pd B

9
8
7

194fv9  d 190-90

10

15         20         25          30

Age    d

Figure 26. Lymphoid leukaemia (ICD6 204.0; ICD7 204.0,204.3; ICD8&9 204)

5

'   . ,  :s ,  .

,704

1960     1970:    1960     1

Ywrd

600

lam

*....           . - :

1 _ ~~~~~~~3

196Oi     9Wh6O-  -1| -970  1960    1990

*;l ee

* 4. . . .. .

* saiz_6 A_ ____

w-W|E-s

,, .. . S

; . . . s

300                               70 8 .0 ;- ...  . .   ........., . .

250                                         2 /t0

1r 1

110020

160                                          Im__ _ _ _ _ _                    36__ _ _9_

50          5'0~T                             ~ -

1960  19                0  1    19                9     P     7       1986  1990
Figure 27. Cancer of unspecified site (ICD6&7 156, 165, 198, 199; ICD8 195-199; ICD9 155.2, 195-199)

.700
-600

50I:

*'5

t 1 ......

0-
-960

7044
D,69

..M.M-                                                          v .          . . ?. 1.       . . .

"          Mm"            .1          -     ... .--.
1.m                                            III      -   ?'   "'   -   -.:

u s s w       *..

70-
./   -~-.   g. .  8 6-

A-A-.- A ah2'

l   . .I

i                          i                         i

Lung

4

aI 4

1960

1950   1960    1970   190    1990

. .. .  .  .   ..f  I

*   r i  . I   - . :  . . .

*      i     ,    d

zl*-

ISO_   _

YU. T            CoA on_
601-

30~~~~~~~~Mdo

01-

1960

1970

1980

1990

1960

1960      1970      1980     1990

.. .  ..:d. . h

.  I

*h     ,~~~~~I I--1

Lung

Colon & Rectm

*CevIx Uei

@ @ ..-.tmsh

1970

1980

1990

Yew of dlnclnc

Yow d I _idene

Figure 28. Mortality 1953-93 and incidence 1960-90, ages 35-69, for the five most commonly fatal/incident cancers in each sex.

210 +

18o+

15 +

120 +

I
8-
1.

I"

I  -.

60 -

K

0

150

I...K

I

S-

a                              I                   I          I                              a

L

I

ni r%

AL-Amik-

i-1, ,       I
I . -.